# Role of TGF‐β1/miR‐382‐5p/SOD2 axis in the induction of oxidative stress in CD34+ cells from primary myelofibrosis

**DOI:** 10.1002/1878-0261.12387

**Published:** 2018-11-16

**Authors:** Chiara Rossi, Roberta Zini, Sebastiano Rontauroli, Samantha Ruberti, Zelia Prudente, Greta Barbieri, Elisa Bianchi, Simona Salati, Elena Genovese, Niccolò Bartalucci, Paola Guglielmelli, Enrico Tagliafico, Vittorio Rosti, Giovanni Barosi, Alessandro M. Vannucchi, Rossella Manfredini, the AGIMM (AIRC‐Gruppo Italiano Malattie Mieloproliferative) investigators

**Affiliations:** ^1^ Department of Life Sciences Centre for Regenerative Medicine University of Modena and Reggio Emilia Italy; ^2^ Department of Experimental and Clinical Medicine CRIMM, Center for Research and Innovation for Myeloproliferative Neoplasms AOU Careggi University of Florence Italy; ^3^ Center for Genome Research University of Modena and Reggio Emilia Italy; ^4^ Center for the Study of Myelofibrosis Laboratory of Biochemistry, Biotechnology and Advanced Diagnostics IRCCS Policlinico San Matteo Foundation Pavia Italy

**Keywords:** miR‐382‐5p, oxidative stress, primary myelofibrosis, reactive oxygen species, superoxide dismutase, TGF‐β1 signaling

## Abstract

Primary myelofibrosis (PMF) is a myeloproliferative neoplasm characterized by an excessive production of pro‐inflammatory cytokines resulting in chronic inflammation and genomic instability. Besides the driver mutations in *JAK2*,*MPL,* and *CALR* genes, the deregulation of miRNA expression may also contribute to the pathogenesis of PMF. To this end, we recently reported the upregulation of miR‐382‐5p in PMF CD34+ cells. In order to unveil the mechanistic details of the role of miR‐382‐5p in pathogenesis of PMF, we performed gene expression profiling of CD34+ cells overexpressing miR‐382‐5p. Among the downregulated genes, we identified superoxide dismutase 2 (*SOD2*), which is a predicted target of miR‐382‐5p. Subsequently, we confirmed miR‐382‐5p/*SOD2* interaction by luciferase assay and we showed that miR‐382‐5p overexpression in CD34+ cells causes the decrease in SOD2 activity leading to reactive oxygen species (ROS) accumulation and oxidative DNA damage. In addition, our data indicate that inhibition of miR‐382‐5p in PMF CD34+ cells restores SOD2 function, induces ROS disposal, and reduces DNA oxidation. Since the pro‐inflammatory cytokine transforming growth factor‐β1 (TGF‐β1) is a key player in PMF pathogenesis, we further investigated the effect of TGF‐β1 on ROS and miR‐382‐5p levels. Our data showed that TGF‐β1 treatment enhances miR‐382‐5p expression and reduces SOD2 activity leading to ROS accumulation. Finally, inhibition of TGF‐β1 signaling in PMF CD34+ cells by galunisertib significantly reduced miR‐382‐5p expression and ROS accumulation and restored SOD2 activity. As a whole, this study reports that TGF‐β1/miR‐382‐5p/SOD2 axis deregulation in PMF cells is linked to ROS overproduction that may contribute to enhanced oxidative stress and inflammation. Our results suggest that galunisertib may represent an effective drug reducing abnormal oxidative stress induced by TGF‐β1 in PMF patients.

**Database linking:**

GEO: https://www.ncbi.nlm.nih.gov/geo/query/acc.cgi?acc=GSE103464.

**Abbreviations**
8‐OH‐dG: 8′hydroxy‐2′deoxyguanosine
ALK5: TGF‐β receptor I kinase
anti‐miR‐Neg: miRNA inhibitor negative control
BM: bone marrow
CB: cord blood
DAPI: 4′,6‐diamidino‐2‐phenylindole
DCF: dichlorofluorescein
GEP: gene expression profile
HSPCs: hematopoietic stem and progenitor cells
IL: interleukin
mimic‐Neg: negative control sample
miRNAs: microRNAs
MPNs: myeloproliferative neoplasms
PB: peripheral blood
PMF: primary myelofibrosis
qRT‐PCR: quantitative RT‐PCR
ROS: reactive oxygen species
RQ: relative quantity
RT: room temperature
SOD2: superoxide dismutase 2
TGF‐β1: transforming growth factor beta 1
WT: wild‐type

## 1 Introduction

Classical Philadelphia‐negative chronic myeloproliferative neoplasms (MPNs) are clonal disorders of hematopoietic stem cell that include essential thrombocythemia (ET), polycythemia vera (PV), and primary myelofibrosis (PMF; Vardiman *et al*., 2009) (Arber *et al*., 2016). MPNs arise due to an acquired stem cell lesion with subsequent clonal expansion leading to proliferation and overproduction of mature myeloid cells. PMF carries the worst prognosis among MPNs with severe clinical hallmarks, including megakaryocyte and granulocyte hyperplasia, splenomegaly due to extramedullary hematopoiesis, and bone marrow (BM) fibrosis (Tefferi, 2005). The mutational landscape of MPNs has been widely studied identifying driver mutations in JAK2, MPL, and CALR genes in 90% of PMF patients (Klampfl *et al.*, 2013; Nangalia *et al.*, 2013; Pardanani *et al.*, 2006). It is known that the mechanism of action of these mutations results in a constitutive activation of the JAK2/signal transducer and activator of transcription (STAT) signaling pathway (Araki *et al*., 2016; Chachoua *et al*., 2016; Marty *et al*., 2016). The JAK/STAT signaling is a central downstream pathway for the majority of the inflammatory cytokines linked to MPNs and their progression (Vainchenker and Kralovics, 2017). Indeed, PMF patients show increased serum level of pro‐inflammatory cytokines (e.g., transforming growth factor‐β1 (TGF‐β1); interleukin (IL)‐8, IL‐12, IL‐15), and excessive production of reactive oxygen species (ROS), which play a central role in the disease evolution (Bjorn and Hasselbalch, 2015; Tefferi *et al*., 2011; Vaidya *et al*., 2012). Several findings support the pathogenic model in which the oxidative stress contributes to a state of chronic inflammation and genomic instability that may give rise to DNA damages promoting disease development and progression (Yahata *et al*., 2011). Moreover, the state of chronic inflammation typical of PMF (Hasselbalch and Bjorn, 2015) is responsible for a self‐perpetuating circle in which inflammation creates ROS that in turn create more inflammation (Gloire *et al*., 2006). Marty *et al*. demonstrated that the inhibition of ROS production could prevent the development of MPNs in JAK2V617F knock‐in mouse model, giving further evidence of the significance of oxidative stress in the pathogenesis of these myeloid malignancies (Marty *et al*., 2013).

Even though extensive studies have provided unambiguous proof of the role of DNA mutations in the pathogenesis of MPNs, the genetic lesions alone do not solely contribute to the full spectrum of the clinical features of PMF, suggesting the existence of unknown genetic or epigenetic cofactors (Chen *et al*., 2010).

In the last decade, growing evidence highlighted that differentially expressed microRNAs (miRNAs) can regulate fundamental processes in hematopoietic stem and progenitor cells (HSPCs), such as proliferation, differentiation, and malignant transformation (Bartel, 2004; Zhan *et al.*, 2013). Of note, miRNA profile studies in MPN patients showed differentially expressed miRNAs among different MPN subtypes (Bruchova *et al*., 2007; Zini *et al*., 2017). Recently, our group identified several differentially expressed genes and miRNAs potentially involved in PMF pathogenesis (Norfo *et al*., 2014). In particular, among the upregulated miRNAs, we detected miR‐382‐5p, known to be associated with other hematologic malignancies, such as acute promyelocytic myeloid leukemia (AML‐M3; Jongen‐Lavrencic *et al*., 2008); (Li *et al*., 2008). In our previous work, we elucidated the role of miR‐382‐5p in normal hematopoiesis and in HSPC fate demonstrating that its enforced expression favors the granulocyte commitment (Zini *et al*., 2016).

Here we focused on the mechanistic role of miR‐382‐5p in the pathogenesis of PMF, by showing its direct involvement in ROS overproduction and oxidative DNA damage induced by the inhibition of its target superoxide dismutase 2 (SOD2).

Then, as TGF‐β1 has been already shown to induce miR‐382‐5p expression in renal cell lines and its role in PMF fibrosis and inflammation has been described (Agarwal *et al.*, 2016; Leask and Abraham, 2004), we investigated whether TGF‐β1 could take part in the overproduction of ROS. In this study, we investigated the link between TGF‐β1 and the overproduction of ROS. The obtained data indicate that the axis TGF‐β1/miR‐382‐5p/SOD2 is responsible at least in part for the enhanced oxidative stress in normal and PMF CD34+ cells, probably contributing to a state of chronic inflammation, that is a typical feature of PMF. Finally, our study demonstrates the efficacy of the inhibitor of TGF‐beta receptor I kinase (TBRI) galunisertib in reducing oxidative stress in PMF CD34+ cells, unveiling a promising therapeutic application of this drug.

## 2 Material and methods

### 2.1 Ethics statement

Human CD34+ cells were purified from umbilical cord blood (CB) samples, collected after normal deliveries, according to the institutional guidelines for discarded material (Clearance of Ethical Committee for Human experimentation of Modena: Secretary office Saverio Santachiara, approval date: 18.01.2005; approval file number # 793/CE).

Primary myelofibrosis CD34+ cells were isolated from the peripheral blood (PB) of three patients in a typical fibrotic phase of the disease according to World Health Organization (WHO) criteria updated in 2016 (Arber *et al*., 2016). The study was performed under the local institutional review board's approved protocol (Florence: approval date: April 22, 2011, approval file number # 2011/0014777; Pavia: approval date: February 24, 2011, file number #174). All subjects provided informed written consent, and the study was carried out in accordance with the Declaration of Helsinki.

### 2.2 CD34+ cell purification and culture

Human CD34+ cells were purified from CB or PB samples as previously described (Bianchi *et al*., 2010; Norfo *et al*., 2014). After immunomagnetic separation, CD34+ cells were seeded into 24‐well plates at 5 × 10^5^ per mL in SYN‐H synthetic serum‐free medium (ABCell‐Bio, Paris, France) supplemented with stem cell factor (SCF; 50 ng·mL^−1^), FLT3‐ligand (FLT3L; 50 ng·mL^−1^), thrombopoietin (TPO; 20 ng·mL^−1^), IL‐6 (10 ng·mL^−1^) and IL‐3 (10 ng·mL^−1^; all from Miltenyi Biotec, Auburn, CA, USA) and electroporated 24 h later.

### 2.3 TGF‐β1 and galunisertib treatment

Purified CB CD34+ cells were seeded into 24‐well plates at 5 × 10^5^ per mL in SYN‐H medium (ABCell‐Bio) without any other supplements and treated with TGF‐β1 (5 ng·mL^−1^; Miltenyi Biotec) for 24 and 48 h. The TGF‐β receptor I kinase (ALK5) inhibitor, galunisertib (LY‐2157299, Catalog #S2230, Aurogene, Rome, Italy), was prepared at 10 mm in dimethyl sulfoxide (DMSO) and stored in aliquots at −20 °C. PMF CD34+ cells were incubated for 24 h with 500 nm galunisertib (Zhou *et al*., 2011).

### 2.4 Electroporation of CD34+ cells

In order to achieve the overexpression of miR‐382‐5p in HSPCs, CB CD34+ cells were electroporated using 4D‐Nucleofector System (Lonza Group Ltd, Basel, Switzerland) as described (Norfo *et al*., 2014). Briefly, 24 h after immunomagnetic separation, CD34+ cells were electroporated twice, once every 24 h, with 3 μg (0.214 nm) of mirVana miR‐382‐5p mimic or mirVana miRNA mimic Negative Control (mimic‐Neg; Life Technologies, Carlsbad, CA, USA). Preliminary experiments were carried out using untransfected and MOCK‐transfected samples as additional controls. For each electroporation, 4 × 10^5 ^CD34+ cells were resuspended in 100 μL of P3 Primary Cell Solution (Lonza) containing mimic miRNA, and pulsed with the program DS112. After each transfection, CD34+ cells were transferred into prewarmed fresh SYN‐H medium (ABCell‐Bio) supplemented with SCF (50 ng·mL^−1^), FLT3L (50 ng·mL^−1^), TPO (20 ng·mL^−1^), IL‐6 (10 ng·mL^−1^), and IL‐3 (10 ng·mL^−1^) and maintained in the same culture conditions as described above. In order to estimate the transfection efficiency, we used BLOCK‐IT fluorescent oligo (catalog #13750062, ThermoFisher Inc., Waltham, MA, USA). Briefly, CD34+ cells were nucleofected twice, once every 24 h, with 0.214 nm of BLOCK‐IT fluorescent oligo and FITC fluorescence was detected by flow cytometry at 24 and 48 h after the last nucleofection.

Primary myelofibrosis CD34+ cells underwent the same electroporation protocol DS112 and were nucleofected 3 times, once every 24 h, with 3 μg of mirVana miR‐382‐5p inhibitor or mirVana miRNA Inhibitor Negative Control (anti‐miR‐Neg).

Cells were analyzed 24 and 48 h after the last nucleofection for both cell viability and miR‐382‐5p and *SOD2* expression.

### 2.5 RNA extraction and gene expression profile

miRNeasy micro RNA isolation kit (Qiagen, Hilden, Germany) was used to isolate and purify total RNA containing small RNAs from CD34+ cells, following the manufacturer's instructions. The purity and integrity of RNA samples were determined by using disposable RNA chips (Agilent RNA 6000 Nano LabChip kit) and the Agilent 2100 Bioanalyzer (Agilent Technologies, Waldbrunn, Germany). NanoDrop ND‐1000 spectrophotometer (NanoDrop Technologies, Wilmington, DE, USA) was used to evaluate the RNA sample concentration, while 260/280 and 260/230 nm ratios were used to assess the RNA purity.

Gene expression profiling was performed in triplicate starting from 100 ng of total RNA obtained from three independent experiments. For microarray analysis, cDNA synthesis and biotin‐labeled target synthesis were performed using the GeneAtlas 3′ IVT Plus Reagent Kit according to the standard protocol supplied by Affymetrix (Santa Clara, CA, USA). The HG‐U219 Array Strip (Affymetrix) hybridization, staining, and scanning were performed by using the GeneAtlas Platform.

Gene expression profile (GEP) data were analyzed by partek gs 6.6 Software Package and normalized using the robust multi‐array average (RMA) procedure (Irizarry *et al*., 2003). Differentially expressed genes were selected as the sequences showing a signal‐log ratio average > 0.4 or < −0.4 in the pairwise comparisons between miR‐382‐5p mimic and negative control samples (mimic‐Neg). Microarray data were analyzed with the two‐tailed Student's *t‐*test for comparison of signal averages in paired samples; *P < *0.05 was considered significant. Analysis of variance (anova) module included in partek gs 6.6 Software Package was used for selection of differentially expressed genes shown in the heatmap. Ingenuity pathways analysis (IPA; http://www.ingenuity.com) was used to find the putative miRNA‐mRNA interactions through IPA's MicroRNA Target Filter.

### 2.6 Quantitative RT‐PCR (qRT‐PCR)

Total RNA (50 ng per sample) was reverse‐transcribed to cDNA using the High‐Capacity cDNA Reverse Transcription Kit (Life Technologies), and real‐time PCR was achieved in triplicate with TaqMan gene expression assays and Fast advanced master mix (all reagents from Life Technologies) by using the AB 7900HT Fast Real‐Time PCR System (Applied Biosystems; Guglielmelli *et al*., 2007a,2007b). Relative quantity (RQ) was carried out using the comparative cycle threshold (CT) method using glyceraldehyde‐3‐phosphate dehydrogenase (GAPDH) as the housekeeping gene. To normalize the data, ΔΔCT was calculated for each sample using the mean of its ΔCT values subtracted from the mean ΔCT value measured in the entire population of healthy subjects, considered as a calibrator; the RQ value was expressed as 2^−ΔΔCT^.

Individual miRNA detection was performed in triplicate using the TaqMan MicroRNA assays (Life technologies) by means of qRT‐PCR. Briefly, 5 ng of total RNA was reverse‐transcribed using the TaqMan MicroRNA Reverse Transcription Kit and miRNA‐specific looped primers. miRNA expression RQ data were calculated as reported above, by using U6 snRNA as the housekeeping control (Norfo *et al*., 2014).

### 2.7 Western blotting

Briefly, CB or PMF CD34+ was lysed in 50 mm Tris pH 7.4, 150 mm NaCl, 10 mm KCl, 1 mm EDTA, 20 mm NaF, 5 mm DTT, 0,25% Na deoxycholate, 0,1% Nonidet P‐40 and protease inhibitor (Complete, catalog #1697468, Roche, Milano, Italy). Total cell lysates (30 mg·mL^−1^ for each sample) were resolved by electrophoresis on a 10% SDS/polyacrylamide gel and transferred to nitrocellulose membrane (GE Healthcare, Little Chalfont, UK). The membranes were checked for loading and transfer efficiency by staining with Red Ponceau. Membranes were blocked with 5% BSA in 0,1% Tween‐20 for 1 h at room temperature (RT), washed, and incubated with primary antibodies: (a) mouse monoclonal anti‐SOD2 antibody (1 : 20 000 dilution, SOD‐2 (A‐2) catalog #sc‐133134, Santa Cruz Biotechnology, Inc., Heidelberg, Germany) overnight at 4 °C; (b) rabbit polyclonal anti‐actin antibody (1 : 2000 dilution, Thermo Fisher Scientific, Inc., Waltham, MA, USA, catalog #PA1‐16889) for 1 h at RT. Horseradish peroxidase‐conjugated goat anti‐mouse and anti‐rabbit antibodies (catalog #sc2005, #sc2004 Santa Cruz Biotechnology Inc) were added at 1 : 5000 and 1 : 10 000, respectively, for 1 h at RT. BM Chemiluminescence Blotting Substrate (POD; Roche) visualized proteins. imagej software was used to quantify the protein level.

### 2.8 Luciferase reporter analysis

The 3′‐UTR reporter analysis was performed to verify the real interaction between miR‐382‐5p and its target sequence on SOD2 3′UTR. Briefly, predicted target site of miR‐382‐5p was cloned into the Pme1 and Xbal sites of pmirGLO Dual‐Luciferase miRNA Target Expression Vector (Promega Italia, Milano, Italy). Synthetic oligonucleotides (wild‐type (WT) miRNA‐binding site: 5′AAACTAGCGGCCGCTAGTTCACTTATTTCATAAACAACTTAT3′, 3′TTTGATCGCCGGCGATCAAGTGAATAAAGTATTTGTTGAATAGATC5′) were designed based on TargetScanHuman Database 7.0 sequences and correspond to 16 nucleotides surrounding the miRNA‐binding sites (seed region) sequence that were annealed before ligation into the pmirGlo plasmid. Mutations in the seed region and luciferase reporter assay in K562 cell line have been performed as previously described (Zini *et al*., 2016). Briefly, K562 cells were cotransfected by means of the Amaxa 4D‐NucleofectorTM System with either a miR‐382‐5p or mimic‐Neg at a concentration of 3.6 μm and with either empty vector or miRNA‐binding site‐containing construct (200 ng·sample^−1^). Firefly and Renilla luciferase activities were measured at 36 and 48 h after electroporation using the Dual‐Luciferase Reporter Assay System (Promega), and luminescence was recorded on a GloMax®‐Multi+ Detection System with Instinct Software (Promega), according to the manufacturer's protocol. Data normalization was performed as previously described (Zini *et al*., 2016).

### 2.9 Detection of ROS using CM‐H_2_DCFDA

The redox‐sensitive fluorochrome 5‐(and 6)‐chloromethyl‐2′, 7′‐dichlorodihydrofluorescein diacetate dye (CM‐H_2_DCFDA, Invitrogen, Carlsbad, CA, USA) was used to measure the intracellular ROS. In order to perform dichlorofluorescein (DCF) staining, 24 h after the last nucleofection, CB or PMF CD34+ cells were loaded with 2 μm CM‐H_2_DCFDA for 20 min at 37 °C.

Before flow cytometric analysis, the cells were removed from loading buffer and incubated in growth medium for 20 min at 37 °C (Marty *et al*., 2013). Data acquisition and analysis were conducted using a BD FACSCanto II (BD Biosciences, San Jose, CA USA). At least 10 000 events were detected for each sample to guarantee a statistical relevance.

### 2.10 Determination of superoxide dismutase activity in CB and PMF CD34+ cells

Superoxide dismutase (SOD) activity was measured 24 h after the last nucleofection using Trevigen's Superoxide Dismutase Assay Kit according to the manufacturer's instructions (Trevigen, Gaithersburg, MD, USA). SOD activity was assayed on 20 μg of total cellular lysate, based on the reduction in nitroblue tetrazolium (NBT) to nitroblue tetrazolium‐diformazan. Since SODs reduce superoxide ion concentration, the reduction in the nitroblue tetrazolium‐diformazan production is a measure of total SOD activity (U·mL^−1^), defined as the amount of enzyme needed to display 50% dismutation of the superoxide radicals. The reduction in nitroblue tetrazolium to nitroblue tetrazolium‐diformazan induced by the superoxide radical ($O_{2}^{-}$ ) was monitored with Beckman Coulter DU®730 Life Science UV/VIS spectrophotometer by reading the absorbance at 550 nm.

### 2.11 Measurement of 8‐OH‐dG level

Oxidative DNA damage was detected in CB and PMF CD34+ cells 24 h after the last nucleofection by measuring the formation of 8′hydroxy‐2′deoxyguanosine (8‐OH‐dG), a ubiquitous marker of oxidative stress. Firstly, DNA was isolated using DNeasy Blood and Tissue Kit (Qiagen, Valencia, CA, USA) and the obtained RNA‐free DNA was used to estimate 8‐OH‐dG levels using a competitive enzyme immunoassay according to the manufacturer's protocol (The OxiSelect**™** Oxidative DNA Damage ELISA Kit, Cell Biolabs, San Diego, CA, USA). 8‐OH‐dG concentration was determined by measuring the absorbance at 450 nm with the Glomax Multi Detection System (Promega, Madison, WI, USA).

### 2.12 Measurement of CB and PMF CD34+ cell viability

Viability measurement was assessed by trypan blue exclusion assay 24 h after the last nucleofection (Humpe *et al*., 2005). In a Neubauer chamber, at least 100 cells were microscopically analyzed in triplicate for cell viability. The mean percentage of living cells of the three analyses was calculated. Furthermore, cell viability was evaluated using XTT Cell Proliferation Assay Kit (Trevigen) in accordance with the manufacturer's instruction. After 24 h from the last nucleofection, CD34+ cells were plated at a density of 50 000 cells/well in 96‐well plates. The optical density at 450 nm was measured at 2, 4, 6, 8, and 24 h by Glomax Multi Detection System (Promega).

### 2.13 SOD2 and Ki‐67 immunofluorescent staining

After 24 h from the last nucleofection, 1 × 10^4^ CB or PMF CD34+ cells were cytocentrifuged, fixed with 4% paraformaldehyde, and permeabilized using 0,2% Triton X‐100 in PBS for 20 min. After blocking with 2% BSA, 0,1% Triton in PBS, slides were incubated with rabbit polyclonal anti‐human Ki‐67 (catalog #ab15580, Abcam, Cambridge, UK) at 1 : 100 dilution in the blocking buffer for 30 min at 37 °C or with mouse monoclonal anti‐SOD2 antibody (catalog #sc‐133134, Santa Cruz Biotechnology Inc.) at 1 : 100 dilution in the blocking buffer overnight at 4 °C. After three washes in PBS, slides were incubated for 30 min at 37 °C with goat anti‐rabbit Alexa Fluor 568‐conjugated secondary antibody at 1 : 2000 dilution (catalog #A‐11011 Invitrogen) or goat anti‐mouse Alexa Fluor 488‐conjugated secondary antibody at 1 : 1000 dilution (catalog # A‐11004 Invitrogen). The slides were rinsed again, and 4′,6‐diamidino‐2‐phenylindole (DAPI) solution was applied for 3 min for nuclear counterstaining. Fluorescent imaging was performed using a high‐resolution Zeiss LSM 510 Meta Confocal fluorescence microscope (Zeiss, Germany) equipped with axiovision software for image analysis. To ensure random sampling, 20 images/slide were taken and cells positive for Ki‐67 or SOD2 were scored.

### 2.14 Ki‐67 Flow cytometry analysis

Ki‐67 expression was analyzed by flow cytometry. Ki‐67 intracellular staining was performed using FIX & PERM Cell Fixation & Cell Permeabilization Kit (ThermoFisher Scientific Inc., Waltham, MA, USA). Briefly, 24 h after the last nucleofection, 1 × 10^5^ cells were fixed for 15 min at RT in reagent A and subsequently fixed in 100% methanol for 10 min at 4 °C. Then, fixed cells were washed with PBS and permeabilized with reagent B for 30 min at RT with rabbit polyclonal anti‐human Ki‐67 (#ab15580, Abcam) at 1 : 250 dilution. Finally, cells were washed with PBS and incubated with fluorescein isothiocyanate goat anti‐rabbit IgG F(ab0)2 fragment (#F0382, Sigma‐Aldrich, St. Louis, MO, USA) for 30 min at RT. Data acquisition and analysis were performed using a BD FACSCanto II (BD Biosciences). At least 10 000 events were detected for each sample to guarantee a statistical relevance.

### 2.15 Statistical analysis

The statistics used for data analysis were based on two‐tailed Student's *t*‐tests for averages comparison in paired samples. Data were analyzed by using Microsoft Excel (Microsoft Office, 2008 release) and are reported as mean ± standard error of the mean (SEM). *P* < 0.05 was considered significant.

### 2.16 Data accessibility

All microarray data were submitted to the Gene Expression Omnibus repository (http://www.ncbi.nlm.nih.gov/geo). GEP data referring to miR‐382‐5p‐overexpressing CD34+ cells can be downloaded at the link (https://www.ncbi.nlm.nih.gov/geo/query/acc.cgi?acc=GSE103464). GEP data related to PMF CD34+ cells are available as series in the Gene Expression Omnibus repository (GSE41812 and GSE53482).

## 3 Results

### 3.1 Gene expression profile (GEP) of miR‐382‐5p‐overexpressing cells

In order to investigate the possible role of miR‐382‐5p in the pathogenesis of PMF, we performed a GEP in CD34+ cells overexpressing miR‐382‐5p.

**Figure 1:**
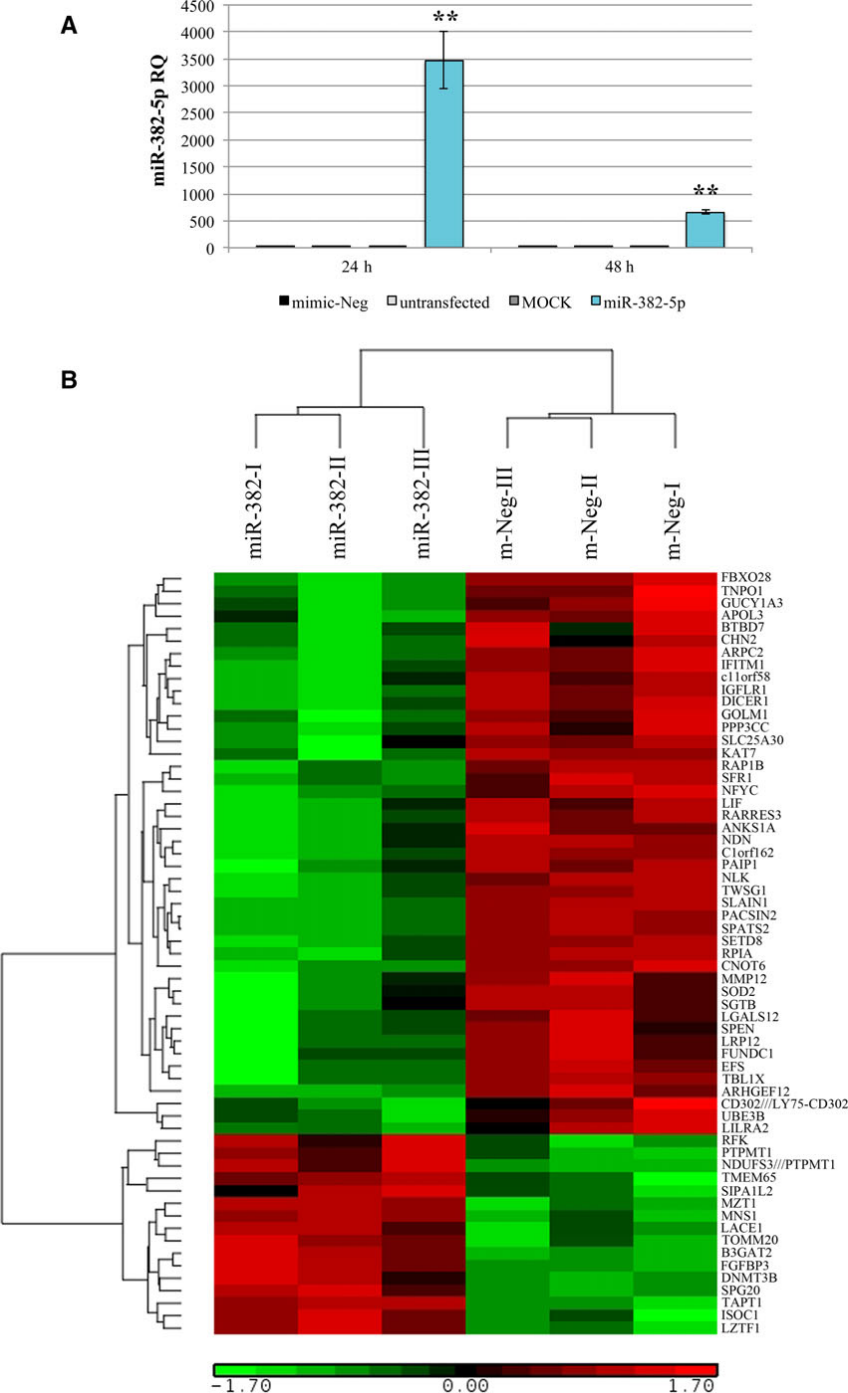
miR‐382‐5p overexpression in CB CD34+ cells. (A) Expression of miR‐382‐5p in CB CD34+ cells was assessed by means of qRT‐PCR at 24 and 48 h after the last nucleofection and data are reported as RQ. U6 snRNA was used as internal controls, and mimic‐Neg was used as reference sample. The results are representative of six independent experiments and are expressed as the mean ± SEM. Two‐tailed Student's *t*‐test: **P < .01 versus mimic‐Neg. (B) GEP of biological triplicates of miR‐382‐5p‐overexpressing cells and mimic‐Neg control samples. The heatmap was computed on a gene list selected by anova using the clustering algorithm included in the Partek GS package (Partek Incorporated, St Louis, MO, USA) by means of Euclidean distance and average linkage. Gene coloring is based on normalized signals, as shown at the bottom of the figure; green indicates reduced expression; red increased expression. Gene symbol is indicated on the right. Mir‐382 and mimic‐Neg groups clustered separately in the dendrogram shown at the top of the heat map.

miRNA overexpression was achieved by nucleofection of miR‐382‐5p mimic. First, we assessed the transfection efficiency using BLOCK‐IT fluorescent oligo and we showed that the proportion of transfected cells is more than 90% (Fig. S1). As shown in Fig. 1A, we obtained a significant overexpression of miR‐382‐5p upon miRNA mimic transfection at 24 and 48 h after the last nucleofection, which was maintained at least until 96 h (data not shown). The expression level of miR‐382‐5p in the three different control samples (i.e., untransfected cells, MOCK‐transfected cells, and cells transfected with miRNA mimic negative control or mimic‐Neg) was not changed (Fig. 1A). Based on these data, we selected the mimic‐Neg sample as negative control and we included it in each experiment.

Microarray analysis was performed 24 h after the last nucleofection, the time point in which miR‐382‐5p expression level achieves the best upregulation in CD34+ cells, as depicted in Fig. 1A. Using the filtering procedure described in Methods, we generated a list of 97 genes significantly modulated in miR‐382‐5p‐overexpressing cells (miR‐382‐5p) compared to mimic‐Neg sample (Table S1). Fig. 1B shows the heatmap of differentially expressed genes further selected by anova (*P* value < 0.05). Among the 75 downregulated genes, we identified *SOD2*, which is one of the most favorable predicted targets of miR‐382‐5p (context score −0.59) according to TargetScanHuman Database (Table S2). Interestingly, GEP analysis of PMF CD34+ cells showed that *SOD2* mRNA is significantly decreased in PMF patients compared to healthy donors (Fig. S2; Norfo *et al*., 2014).

The SODs are a family of antioxidant enzymes that catalyze the dismutation of superoxide radical anions into hydrogen peroxide. In particular, manganese superoxide dismutase (MnSOD‐*SOD2*) has emerged as a crucial tumor suppressor gene and a key mediator in the mitochondrial clearance of detrimental ROS (Van Remmen *et al*., 2003). Since ROS play a crucial role in disease progression of MPNs (Bjorn and Hasselbalch, 2015), we decided to investigate the possible involvement of the miR‐382‐5p/*SOD2* axis in the induction of oxidative stress in normal and PMF CD34+ cells.

### 3.2 *SOD2* is a target of miR‐382‐5p

**Figure 2:**
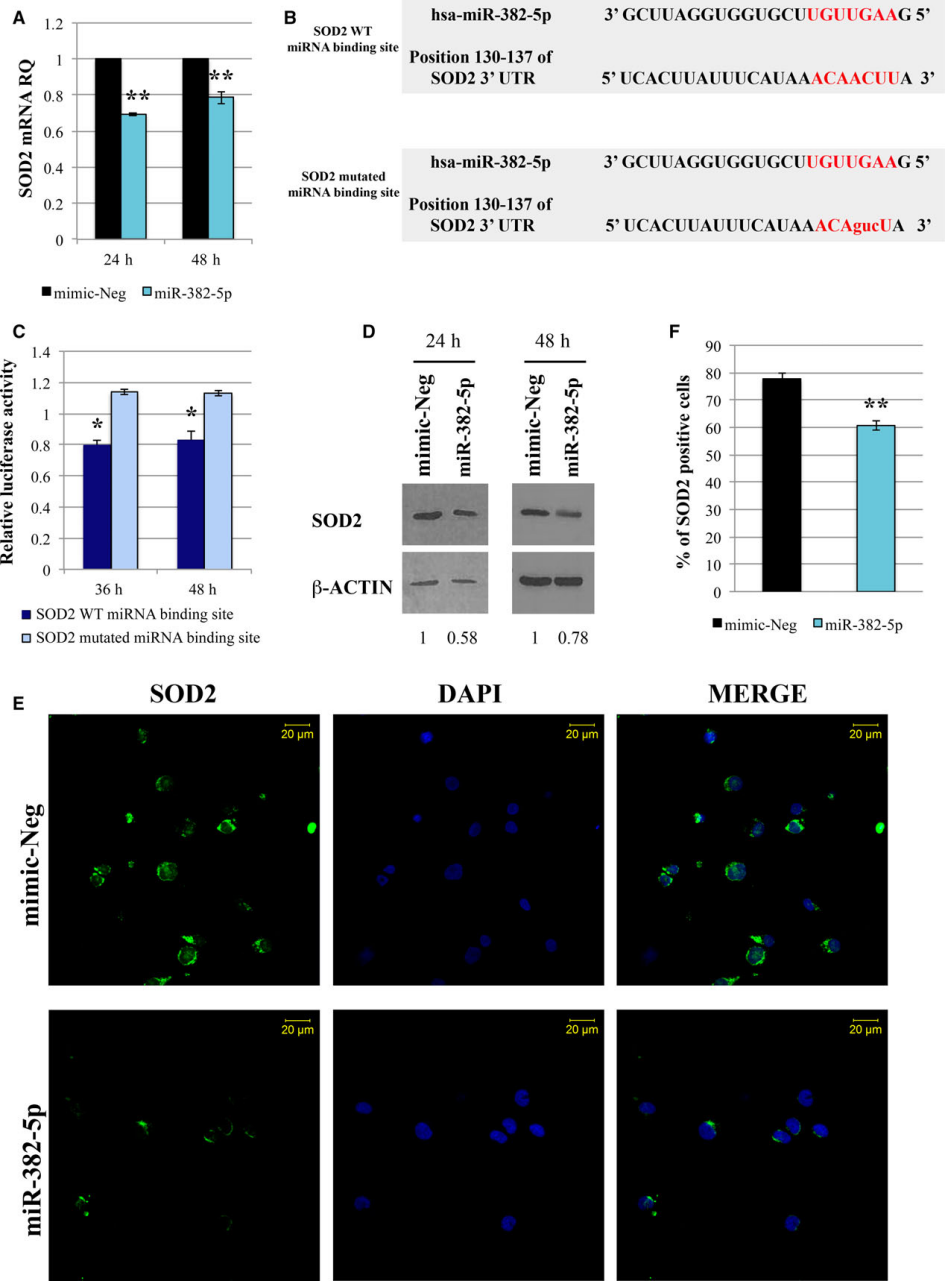
SOD2 is a target of miR‐382‐5p. (A) Expression of *SOD2* mRNA in CB CD34+ cells overexpressing miR‐382‐5p. The *SOD2* level was assessed by qRT‐PCR 24 and 48 h after the last nucleofection. GAPDH was used as housekeeping gene, and mimic‐Neg was used as reference sample. Data are reported as RQ. The results come from six independent experiments. (B) Sequence of oligonucleotides containing the WT miRNA‐binding site in 3′‐UTR of *SOD2* mRNA as predicted by TargetScanHuman Database (Release 7.0) and the mismatched (mutated) version of this target site. miR‐382‐5p seed region is shown in red. Mutated nucleotides are in lowercase. (C) Validation of *SOD2* as miR‐382‐5p target by dual‐luciferase reporter assay. K562 cells were cotransfected with the construct containing the putative WT or mutated miR‐382‐5p binding site. Firefly luciferase activity was measured at 36 and 48 h in K562‐transfected cells, and data were normalized to Renilla luciferase activity. Each bar represents the luciferase activity upon miRNA overexpression normalized on the value of the same 3′‐UTR luciferase vector upon mimic‐Neg transfection. The results come from three independent experiments. (D) Western blot analysis of SOD2 protein level in whole‐cell lysate from CB CD34+ cells transduced with miR‐382‐5p or mimic‐Neg at 24 and 48 h after the last nucleofection. β‐Actin was used as loading control. (E) Representative image of SOD2 immunofluorescence analysis in CB CD34+ cells overexpressing miR‐382‐5p or transfected with mimic‐Neg at 24 h after the last nucleofection. Cells were labeled with anti‐SOD2 antibody (green fluorescence), and nuclear counterstaining was performed with DAPI (blue fluorescence). Scale bar: 20 μm. Original magnification = 40X. To ensure random sampling, 20 images/slide were captured and cells positive for SOD2 were scored. Fluorescence imaging was performed using the Zeiss LSM 510 Meta Confocal Microscope. (F) Quantification of SOD2 positivity in CB CD34+ cells overexpressing miR‐382‐5p compared to negative control. At least 200 cells for each experiment were scored. The results come from six independent experiments. Values are reported as mean ± standard error of the mean (SEM). Two‐tailed Student's *t*‐test: **P < .01 versus mimic‐Neg; *P < .05 versus mimic‐Neg.

In order to validate the negative correlation between miR‐382‐5p and *SOD2* expression in CD34+ cells, we measured the level of *SOD2* in miR‐382‐5p‐overexpressing cells. As shown in Fig. 2A, *SOD2* mRNA is significantly downregulated upon miR‐382‐5p overexpression at 24 and 48 h after the last nucleofection. Then, to confirm that *SOD2* is a real target of miR‐382‐5p, we performed a luciferase reporter assay, by cloning the predicted miRNA‐binding site of *SOD2* downstream of firefly luciferase gene in Dual Luciferase pmirGLO vector (Fig. 2B). Transient cotransfection of either miR‐382‐5p or mimic‐Neg with WT or mutated miRNA‐binding site was performed in K562 cells. As shown in Fig. 2C, luciferase activity is significantly decreased by miR‐382‐5p overexpression when K562 cells were transfected with the construct containing the WT miRNA‐binding site of *SOD2*. Mutations in the seed region‐binding side abrogate the effect of miR‐382‐5p overexpression. Furthermore, we confirmed that miR‐382‐5p overexpression induces a reduction in SOD2 protein level at 24 and 48 h after the last nucleofection (Fig. 2D). Immunofluorescence for SOD2 showed a significant reduction in SOD2 in miR‐382‐5p‐overexpressing cells compared to mimic‐Neg (Fig. 2E,F).

### 3.3 miR‐382‐5p overexpression reduces SOD2 activity, and increases ROS production and oxidative DNA damage

**Figure 3:**
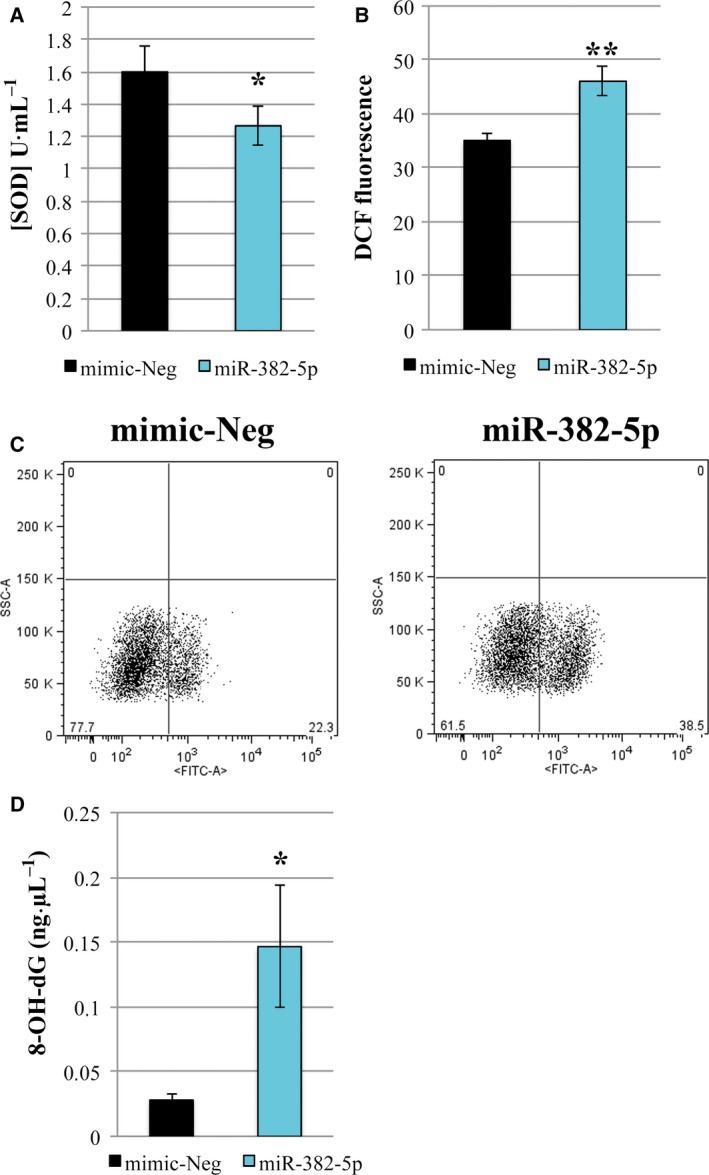
mir‐382‐5p overexpression in CB CD34+ cells reduces SOD activity, and increases ROS production and oxidative DNA damage. (A) Effect of miR‐382‐5p overexpression on SOD activity (U·mL^−1^) determined 24 h after the last nucleofection. (B) The intracellular level of ROS in miR‐382‐5p and mimic‐Neg was measured using DCF staining by flow cytometric analysis 24 h after the last nucleofection. (C) Representative flow cytometry dot plots showing the side scatter (SSC) versus DCF fluorescence distribution in miR‐382‐5p‐overexpressing cells and mimic‐Neg sample at 24 h after the last nucleofection. (D) The level of 8‐OH‐dG in miR‐382‐5p and mimic‐Neg was measured at 24 h after the last nucleofection. The results are representative of six independent experiments and are expressed as the mean ± SEM. Two‐tailed Student's *t*‐test: **P < .01 versus mimic‐Neg; *P < .05 versus mimic‐Neg.

Superoxide dismutase 2 is the major mitochondrial enzyme responsible for superoxide dismutation, preventing the production and accumulation of potentially damaging ROS. For this reason, we wondered whether miR‐382‐5p overexpression could affect SOD2 activity by assaying for superoxide dismutase reaction using Trevigen's Superoxide Dismutase Assay Kit. Fig. 3A shows that the SOD activity is significantly decreased in miR‐382‐5p‐overexpressing cells compared to negative control. Then, by using CM‐H_2_DCFDA staining, we detected an accumulation of ROS in miR‐382‐5p‐overexpressing CD34+ cells (Fig. 3B,C). Since the enhanced production of ROS can lead to DNA damage in hematopoietic stem cells (Yahata *et al*., 2011), we measured DNA 8‐OH‐dG level, a known marker of oxidative stress‐mediated DNA damage. As shown in Fig. 3D, we demonstrated a significant increase in 8‐OH‐dG level in cells overexpressing miR‐382‐5p compared to negative control.

These results suggest that miR‐382‐5p, through the inhibition of its target *SOD2,* causes an increase in intracellular ROS level, which in turn leads to the DNA damage.

### 3.4 miR‐382‐5p overexpression increases proliferation of CD34+ cells

Since *SOD2* has been already described as a tumor suppressor gene and an inverse correlation between its expression level and proliferation rate has been already reported in HSPCs (Hurt *et al*., 2007), we investigated the role of miR‐382‐5p in CD34+ cell proliferation.

**Figure 4:**
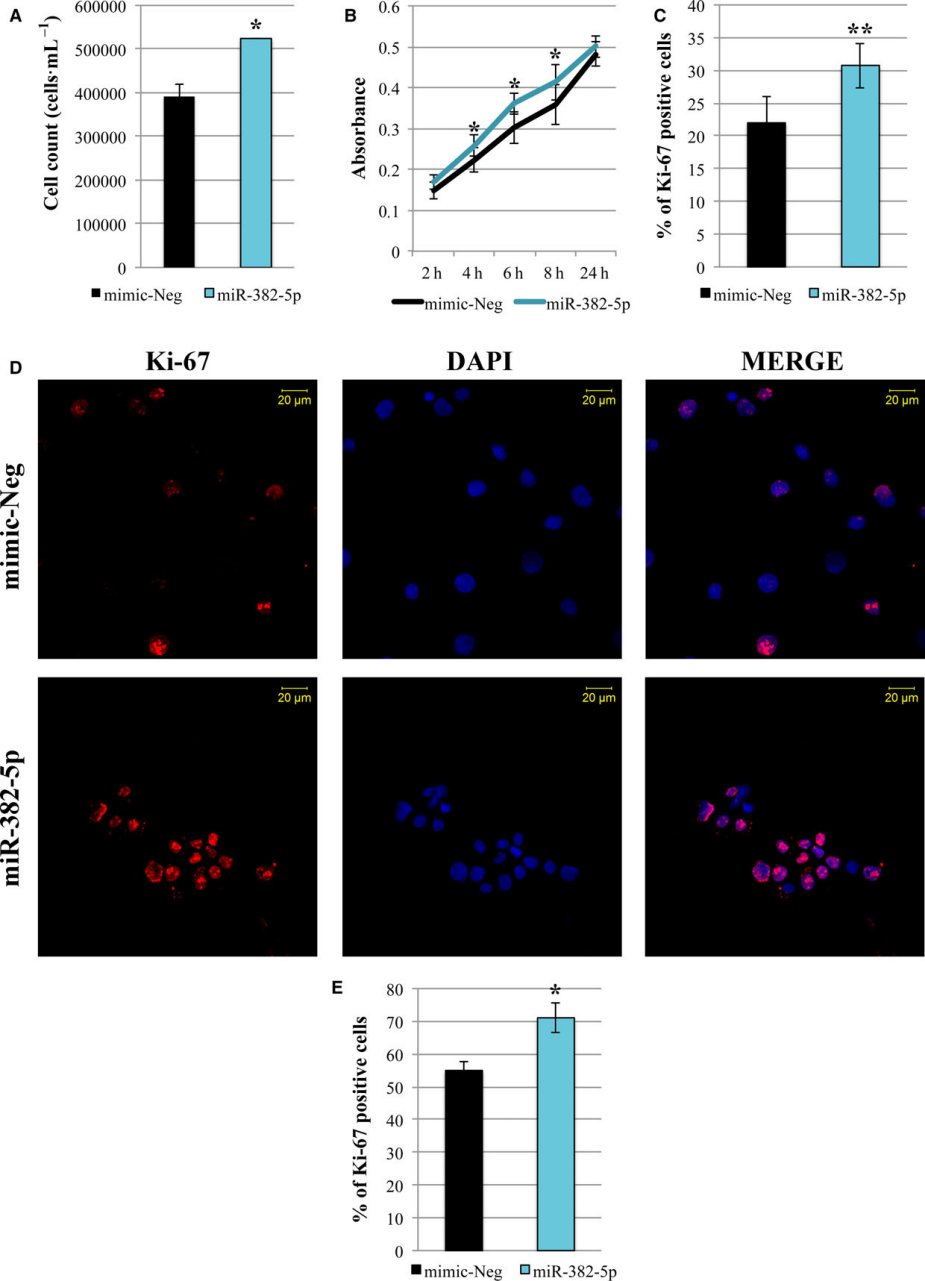
miR‐382‐5p overexpression increases proliferation of CB CD34+ cells. (A) Viability measurement was assessed by cell count (cells·mL^−1^) 24 h after the last nucleofection by trypan blue exclusion assay. (B) The proliferation rate was evaluated at 24 h after the last nucleofection using XTT Cell Proliferation Assay (Trevigen). CD34+ cells were plated at a density of 50 000 cells/well in a 96‐well plate. The absorbance at 450 nm was monitored at different time points. (C) Flow cytometric analysis to estimate the number of proliferating cells using Ki‐67‐antibody. The histogram shows the percentage of Ki‐67‐positive cells in miR‐382‐5p sample compared to negative control at 24 h after the last nucleofection. (D) Representative image of Ki‐67 immunofluorescence analysis in miR‐382‐5p and mimic‐Neg transfected CD34+ cells at 24 h after the last nucleofection. Cells were labeled with anti‐Ki‐67 antibody (red fluorescence), and nuclear counterstaining was performed with DAPI (blue fluorescence). Scale bar, 20 μm. Original magnification = 40X. To ensure random sampling, 20 images/slide were captured and cells positive for Ki‐67 were scored. Fluorescence imaging was performed using the Zeiss LSM 510 Meta Confocal Microscope. (E) Quantification of Ki‐67 positivity in CD34+‐overexpressing cells compared to negative control. At least 200 cells for each experiment were scored. The results are representative of three independent experiments and are expressed as the mean ± SEM. Two‐tailed Student's *t*‐test: **P < .01 versus mimic‐Neg; *P < .05 versus mimic‐Neg.

First of all, trypan blue exclusion assay highlighted a significant increase in cell count in miR‐382‐5p compared to mimic‐Neg sample (Fig. 4A). Moreover, as shown in Fig. 4B, the effect of miR‐382‐5p on cell viability was further confirmed by XTT assay. Accordingly, flow cytometry analysis showed that cells overexpressing miR‐382‐5p display a high positivity for Ki‐67 antigen, expressed in all proliferating cells (Fig. 4C; Whitfield *et al*., 2006); (Sobecki *et al*., 2016). Finally, the immunofluorescence staining using an anti‐Ki‐67 antibody demonstrated that miR‐382‐5p overexpression leads to an increased percentage of Ki‐67‐positive cells (Fig. 4D–E).

Altogether, these results suggest a positive effect of miR‐382‐5p on CD34+ cell proliferation.

### 3.5 Inhibition of miR‐382‐5p reduces oxidative stress and decreases cell proliferation of PMF CD34+ cells

**Figure 5:**
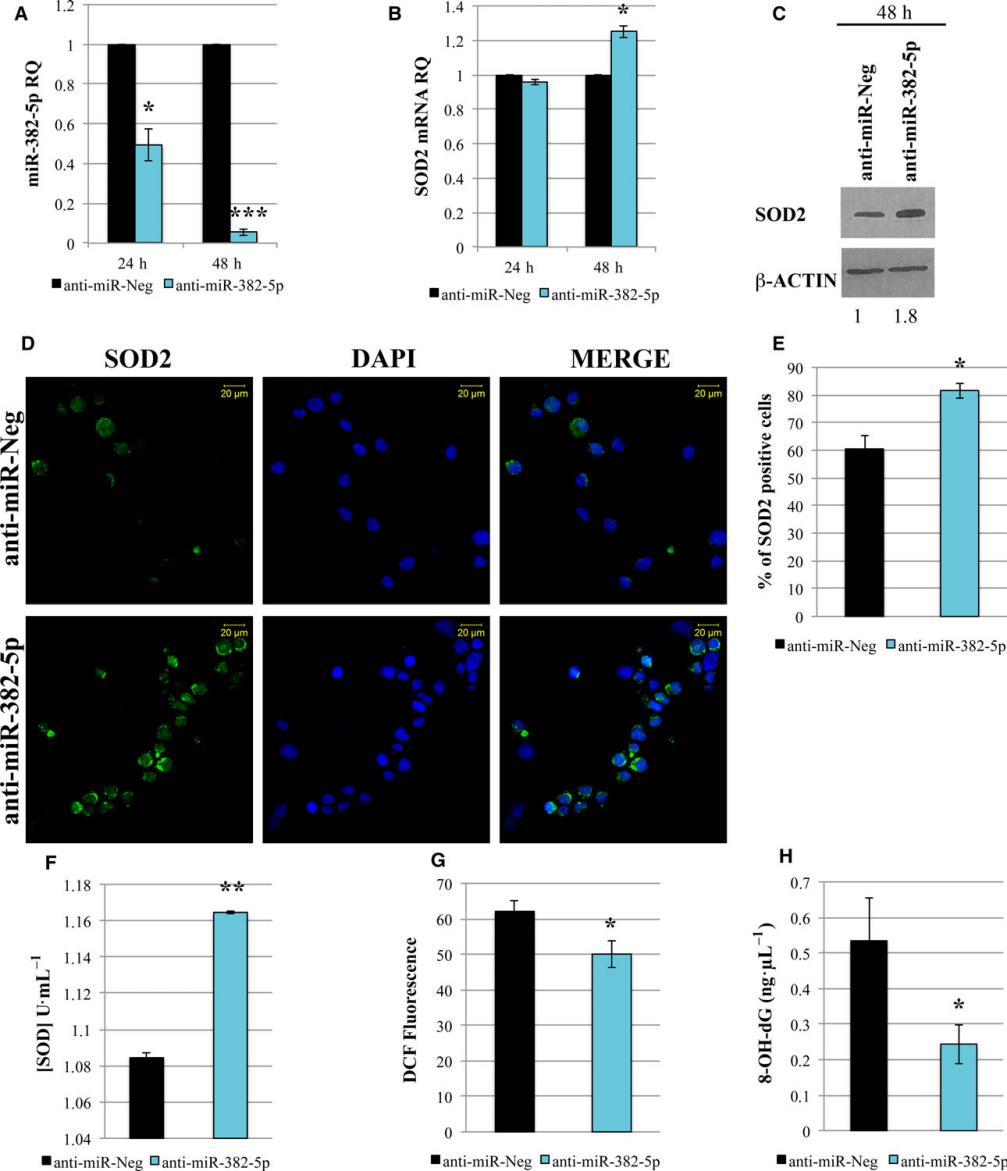
Inhibition of miR‐382‐5p reduces oxidative stress in PMF CD34+ cells. (A,B) Expression level of miR‐382‐5p and *SOD2* in PMF CD34+ cells 24 and 48 h after the last nucleofection by means of qRT‐PCR. Data are reported as RQ. U6 snRNA and GAPDH were used as internal controls, and anti‐miR‐Neg was used as calibrator sample. (C) Immunoblotting of SOD2 protein level in lysates from anti‐miR‐Neg and anti‐miR‐382‐5p transfected PMF CD34+ cells at 48 h after the last nucleofection. β‐actin protein level is reported as loading control. (D) Representative image of immunofluorescence analysis using SOD2‐antibody (green fluorescence) in PMF CD34+ cells transfected with miR‐382‐5p inhibitor or anti‐miR‐Neg at 48 h after the last nucleofection. Nuclear counterstaining was performed with DAPI (blue fluorescence). Scale bar, 20 μm. Original magnification = 40X. To ensure random sampling, 20 images/slide were captured and cells positive for SOD2 were scored. Fluorescence imaging was performed using the Zeiss LSM 510 Meta Confocal Microscope. (E) Quantification of SOD2 positivity in PMF CD34+ cells after miRNA inhibition. At least 200 cells for each experiment were scored. (F) Effect of miR‐382‐5p inhibition on SOD activity (U·mL^−1^) determined 48 h after the last nucleofection. (G) Quantification of intracellular ROS level measured by DCF staining 48 h after the last nucleofection. (H) ROS‐induced DNA damage was measured through the level of 8‐OH‐dG at 48 h after the last nucleofection. Values are reported as mean ± standard error of the mean (SEM). Two‐tailed Student's *t*‐test: ***P < .001 versus anti‐miR‐Neg **P < .01 versus anti‐miR‐Neg; *P < .05 versus anti‐miR‐Neg.

In order to clarify whether miR‐382‐5p could be a key player in PMF pathogenesis by promoting ROS overproduction in hematopoietic progenitors, we inhibited miR‐382‐5p in PMF CD34+ cells by transient transfection of mirVana miRNA inhibitor. As shown in Fig. 5A, we obtained a significant reduction in miR‐382‐5p expression level at 24 and 48 h after the last nucleofection. At the same time points, we evaluated *SOD2* expression, demonstrating a relevant increase in *SOD2* at both mRNA and protein level at 48 h after last nucleofection (Fig. 5B,C). These results were confirmed by SOD2 immunofluorescence analysis that shows a strong increase in protein expression in PMF CD34+ cells upon miR‐382‐5p downregulation (Fig. 5D–E). Moreover, miR‐382‐5p inhibition restores SOD activity (Fig. 5F), causes a decrease in ROS accumulation (Fig. 5G), and leads to a significant reduction in oxidative stress‐mediated DNA damage (Fig. 5H).

**Figure 6:**
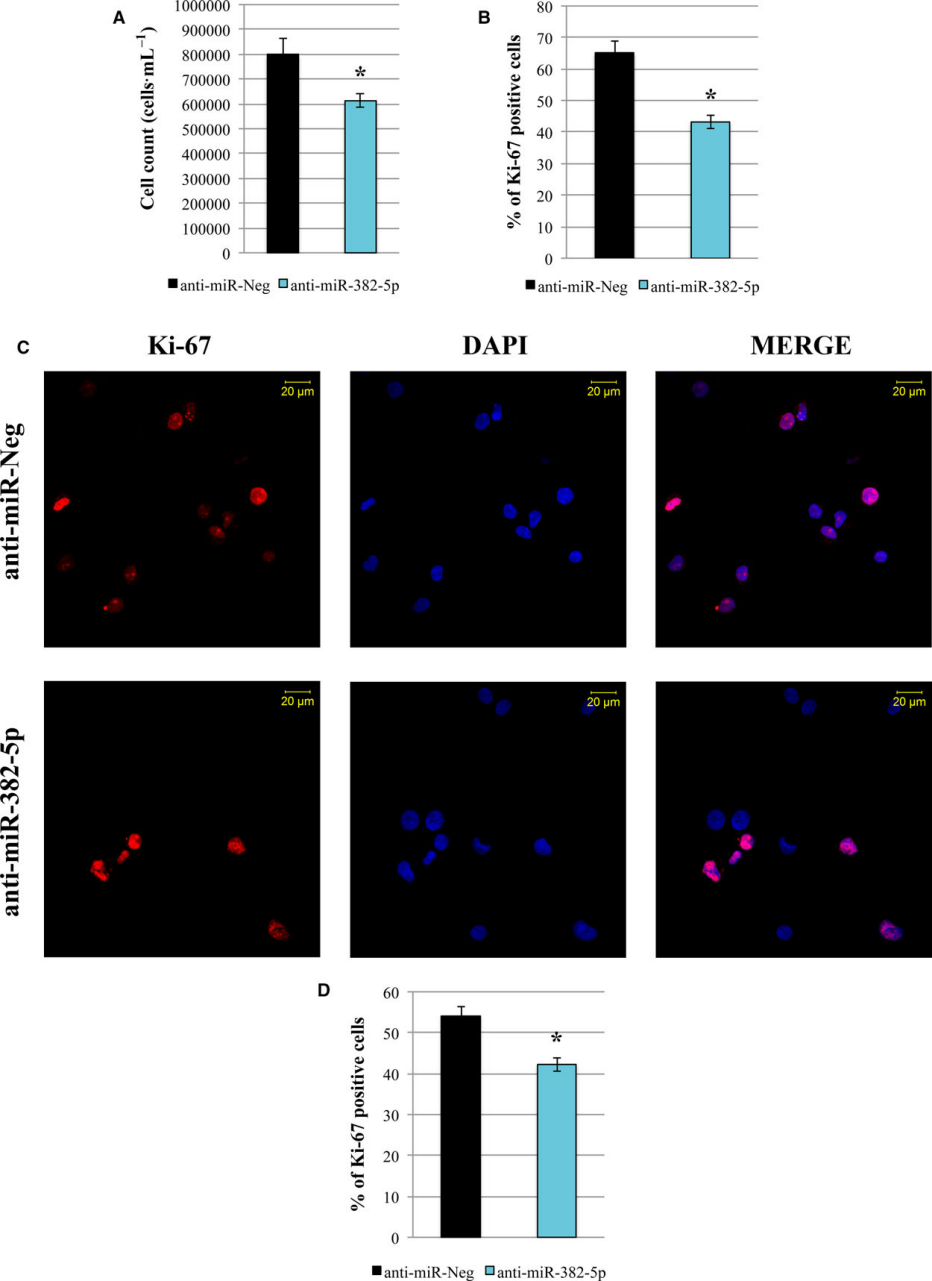
Effect of miR‐382‐5p inhibition on PMF CD34+ cell proliferation. (A) Viability measurement was assessed by cell count (cells·mL^−1^) 48 h after the last nucleofection by trypan blue exclusion assay. (B) Percentage of Ki‐67‐positive cells measured by flow cytometry at 48 h after the last nucleofection. (C) Representative image of Ki‐67 immunofluorescence analysis in PMF CD34+ cells transfected with miR‐382‐5p inhibitor or anti‐miR‐Neg at 48 h after the last nucleofection. Cells were labeled with anti‐Ki‐67 antibody (red fluorescence), and nuclear counterstaining was performed with DAPI (blue fluorescence). Scale bar: 20 μm. Original magnification = 40X. To ensure random sampling, 20 images/slide were captured and cells positive for SOD2 were scored. Fluorescence imaging was performed using the Zeiss LSM 510 Meta Confocal Microscope. (D) Quantification of Ki‐67 positivity in PMF CD34+ cells transfected with miR‐382‐5p inhibitor compared to negative control. At least 200 cells for each experiment were scored. The results are representative of three independent experiments and are expressed as the mean ± SEM. Two‐tailed Student's *t*‐test: *P < .05 versus anti‐miR‐Neg.

Interestingly, trypan blue exclusion assay highlighted a significant decrease in cell count upon miR‐382‐5p inhibition (Fig. 6A). Furthermore, flow cytometry analysis for Ki‐67 revealed that miR‐382‐5p downregulation impairs the ability of PMF CD34+ cells to proliferate (Fig. 6B). These results were further confirmed by immunofluorescence analysis for the detection of Ki‐67 antigen, which shows a reduction in proliferating Ki‐67‐positive cells after miR‐382‐5p inhibition (Fig. 6C,D). These data suggested that miR‐382‐5p inhibition could impair the proliferation capability of PMF CD34+ cells.

### 3.6 Transforming Growth Factor‐β1 induces ROS production in CB CD34+ cells through the modulation of miR‐382‐5p/*SOD2* axis

Transforming growth factor beta 1 is a key mediator of inflammation and fibrosis, widely described as involved in PMF pathogenesis (Agarwal *et al*., 2016) and able to upregulate miR‐382‐5p in renal fibrosis (Kriegel *et al*., 2010). For this reason, we decided to study the axis TGF‐β1/miR‐382‐5p/*SOD2* in CB CD34+ cells in order to understand whether it could be involved in the overproduction of ROS.

**Figure 7:**
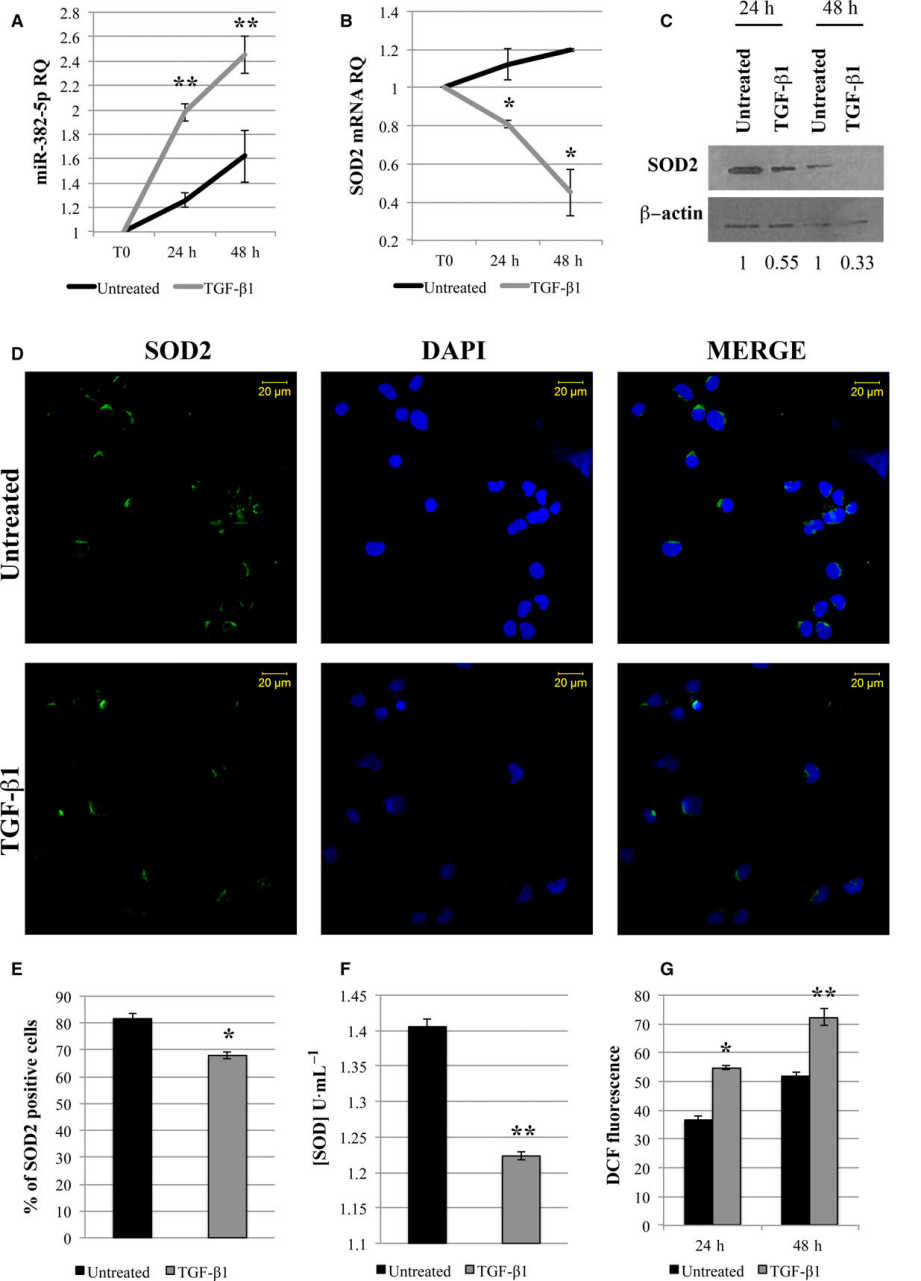
Transforming Growth Factor‐β1 induces ROS production in CB CD34+ cells through the modulation of miR‐382‐5p/*SOD2* axis. (A,B) CB CD34+ cells were treated with 5 ng·mL^−1^ of TGF‐β1 for 48 h. qRT‐PCR analysis was performed to evaluate the expression level of miR‐382‐5p and *SOD2* upon TGF‐β1 treatment at different time points: before treatment (T0), 24, and 48 h. U6 snRNA and GAPDH were used as internal controls, and untreated sample was used as calibrator. (C) SOD2 protein level was evaluated by western Blot analysis in lysates from TGF‐β1‐treated and untreated cells 24 and 48 h after treatment. β‐Actin protein level is reported as loading control. (D) Immunofluorescence analysis of SOD2 (green fluorescence) in TGF‐β1‐treated and untreated cells at 24 h after treatment. Nuclear counterstaining was performed with DAPI (blue fluorescence). Scale bar, 20 μm. Original magnification = 40X. To ensure random sampling, 20 images/slide were captured and cells positive for SOD2 were scored. Fluorescence imaging was performed using the Zeiss LSM 510 Meta Confocal Microscope. (E) Quantification of SOD2 positivity in CD34+ treated with TGF‐β1 compared to untreated cells. At least 200 cells for each experiment were scored. (F) TGF‐β1 effect on SOD activity (U·mL^−1^) at 24 h after treatment. (G) ROS level measured by DCF staining 24 and 48 h after TGF‐β1 treatment. The results are representative of three independent experiments and are expressed as the mean ± SEM. Two‐tailed Student's *t*‐test: **P < .01 versus untreated cells; *P < .05 versus untreated cells.

To this end, we examined the expression level of both miR‐382‐5p and *SOD2* at 24 and 48 h after TGF‐β1 treatment. As shown in Fig. 7A, real‐time PCR analysis reveals a strong induction of miR‐382‐5p in TGF‐β1‐treated CD34+ cells compared to untreated cells. Conversely, we observe a decrease in *SOD2* mRNA at both 24 and 48 h after TGF‐β1 treatment (Fig. 7B). The suppression of SOD2 protein level was further confirmed by western blot analysis and *in situ* immunofluorescence (Fig. 7C–E). Moreover, we demonstrated that TGF‐β1 treatment induces a significant reduction in SOD activity and a remarkable induction of ROS production (Fig. 7F,G).

Overall, these data suggest a direct involvement of TGF‐β1 in ROS accumulation through the modulation of miR‐382‐5p/*SOD2* axis.

### 3.7 Galunisertib (LY‐2157299) reduces ROS production in PMF CD34+ cells

**Figure 8:**
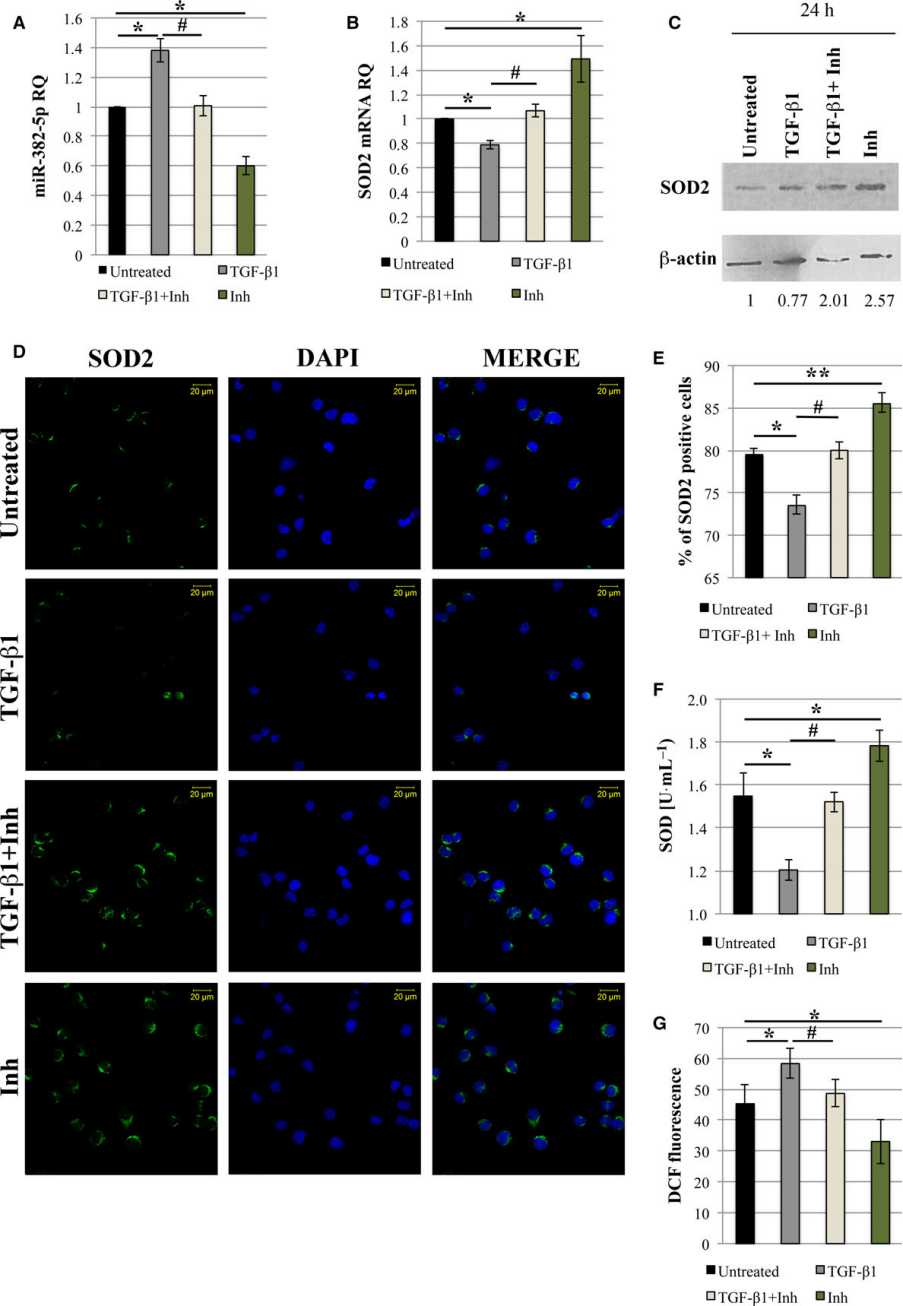
Galunisertib (LY‐2157299) restores SOD2 activity and reduces ROS accumulation in PMF CD34+ cells. PMF CD34+ cells were treated with TGF‐β1 or galunisertib (Inh) alone or TGF‐β1 and galunisertib (TGF‐β1+Inh) in combination. Untreated cells were used to normalize the data. (A,B) qRT‐PCR analysis was performed to evaluate the expression level of miR‐382‐5p and *SOD2* at 24 h after treatments. U6 snRNA and GAPDH were used as internal controls. (C) Western blot analysis was performed after 24 h of treatment on the whole‐cell lysate from TGF‐β1/TGF‐β1+Inh/Inh‐treated and untreated cells. β‐actin protein level is reported as loading control. (D) Immunofluorescence analysis of SOD2 (green fluorescence) in TGF‐β1/TGF‐β1+Inh/Inh‐treated and untreated cells at 24 h after treatments. Nuclear counterstaining was performed with DAPI (blue fluorescence). Scale bar, 20 μm. Original magnification = 40X. To ensure random sampling, 20 images/slide were captured and cells positive for SOD2 were scored. Fluorescence imaging was performed using the Zeiss LSM 510 Meta Confocal Microscope. (E) Quantification of SOD2 positivity. At least 200 cells for each experiment were scored. (F) SOD activity (U·mL^−1^) has been determined after 24 h of TGF‐β1/TGF‐β1+Inh/Inh treatments and compared to untreated cells. (G) Level of ROS production measured by DCF staining 24 h after TGF‐β1/TGF‐β1+Inh/Inh treatment. The results are representative of three independent experiments and are expressed as the mean ± SEM. Two‐tailed Student's *t*‐test: **P < .01 versus untreated cells; *P < .05 versus untreated cells. ^#^ *P* < 0.05 versus TGF‐β1‐treated cells. Inh, inhibitor (galunisertib).

Since we have shown the significant role of TGF‐β1 in the induction of oxidative stress in normal CD34+ cells, we further tested the potential efficacy of galunisertib, an ALK5 inhibitor in PMF CD34+ cells. Firstly, we assessed the expression level of miR‐382‐5p and *SOD2* after treatment with TGF‐β1 or galunisertib alone or in combination in PMF CD34+ cells. According to results obtained in normal CD34+ cells, the expression level of miR‐382‐5p is significantly enhanced in TGF‐β1‐treated cells (Fig. 8A); conversely, *SOD2* mRNA is reduced after TGF‐β1 treatment (Fig. 8B). Galunisertib treatment seems to restore the expression of both miR‐382‐5p and SOD2 in TGF‐β1‐treated cells. In particular, in the presence of galunisertib alone, miR‐382‐5p expression is suppressed, whereas *SOD2* mRNA is significantly increased compared to untreated cells (Fig. 8A,B). Western blot analysis and immunofluorescence further confirmed these results (Fig. 8C–E). Interestingly, SOD activity is restored in PMF CD34+ cells stimulated by TGF‐β1 upon treatment with galunisertib. Moreover, we observed that galunisertib alone is able to restore the basal SOD activity in PMF CD34+ cells (Fig. 8F).

According to our previous results, flow cytometric analysis of ROS content shows an increase in ROS accumulation upon TGF‐β1 treatment, while we observe a reduction in ROS level in TGF‐β1 stimulated cells in the presence of galunisertib. Remarkably, the effect is also evident when PMF CD34+ cells are treated with galunisertib alone (Fig. 8G).

Interestingly, in normal CD34+ cells we observed that the treatment with galunisertib alone did affect neither miR‐382‐5p/SOD2 expression (Fig. S3A,B) nor ROS production (Fig. S3C). However, galunisertib was able to revert the effects on miR‐382‐5p/SOD2 expression levels and ROS production in TGF‐β1‐stimulated CB CD34+ cells (Fig. S3).

Altogether, these results demonstrate that galunisertib is able to abrogate the TGF‐β1‐driven induction of oxidative stress in PMF CD34+ cells, pointing out the importance of the axis TGF‐β1/miR‐382‐5p/*SOD2* in PMF pathogenesis.

## 4 Discussion

In the last decades, growing evidence has highlighted the role of deregulated miRNA expression in MPN pathogenesis. In order to identify a series of miRNAs and coding genes potentially involved in PMF pathogenesis, we recently obtained gene and miRNA expression profiles of CD34+ HSPCs from PMF patients. Integrative analysis of gene and miRNA expression profiles highlighted miR‐382‐5p among miRNAs significantly upregulated in PMF CD34+ cells (Norfo *et al*., 2014). Of note, miR‐382‐5p deregulated expression has been previously reported in other hematological malignancies, such as acute promyelocytic myeloid leukemia (AML‐M3; Jongen‐Lavrencic *et al*., 2008). In addition, we unveiled the key role of miR‐382‐5p in HSPC fate decision toward granulocyte lineage (Zini *et al*., 2016).

In order to shed light on the role of miR‐382‐5p in PMF pathogenesis, we assessed the gene expression changes induced by miR‐382‐5p overexpression in normal CD34+ cells. Among genes downregulated after miR‐382‐5p overexpression, we selected *SOD2*, which is associated with the most favorable prediction score according to TargetScanHuman prediction algorithm. Interestingly, *SOD2* expression is decreased in PMF CD34+ cells compared to healthy subjects (Norfo *et al*., 2014).

The SOD antioxidant family is responsible for the dismutation of the superoxide radical anion in hydrogen peroxide and provides an essential defense against the harmful effect of ROS (Szeto, 2006). High levels of ROS are known to be involved in the pathogenesis of several solid cancers and hematological malignancies (Hurt *et al.*, 2007; Waris and Ahsan, 2006; Zheng *et al.*, 2013) and to play an important role in the initiation and progression of MPNs (Bjorn and Hasselbalch, 2015; Vener *et al*., 2010). Indeed, as previously reported by Vener *et al*., MPN patients show significantly higher serum ROS level if compared to healthy donors (Vener *et al*., 2010). Among SOD family members, manganese superoxide dismutase 2 (MnSOD‐*SOD2*) plays an essential role not only in cancer but also in a wide range of stress‐induced diseases due to its localization in the mitochondria, where the production of ROS is higher compared to the other cellular compartments (Inoue *et al*., 2003). In particular, *SOD2* has been described as a tumor suppressor gene (Van Remmen *et al*., 2003) and its decreased expression level has been reported in a wide variety of diseases (Wang *et al*., 2016) (Hitchler *et al*., 2008), including hematological malignancies (Hurt *et al*., 2007).

For this reason, we decided to investigate the potential involvement of the axis miR‐382‐5p/*SOD2* in the induction of oxidative stress in normal and PMF CD34+ cells. Firstly, we demonstrated that *SOD2* is a real target of miR‐382‐5p by means of luciferase reporter assay. According to these data, we observed a significant downregulation of *SOD2* at both mRNA and protein level upon miR‐382‐5p overexpression in CD34+ cells. Furthermore, we showed that enforced miR‐382‐5p expression leads to a reduction in SOD activity, which in turn determines a significant ROS accumulation and the consequent increase in DNA oxidation.

Moreover, we demonstrated that miR‐382‐5p overexpression stimulates CD34+ cell proliferation. Since elevated ROS level has been reported to promote mitosis in CD34+ cells (Hole *et al*., 2010), and enhanced SOD2 activity has been associated with proliferation inhibition in several cell lines (Oberley, 2005), we hypothesized that miR‐382‐5p could act on cell proliferation through the modulation of *SOD2* expression and ROS overproduction.

As previously reported by Norfo *et al*., while miR‐382‐5p expression is increased, *SOD2* mRNA is decreased in PMF CD34+ cells (Norfo *et al*., 2014). Therefore, in order to confirm that miR‐382‐5p is a key player in PMF pathogenesis, we performed silencing experiments in PMF CD34+ cells. Our results show that miR‐382‐5p downregulation restores SOD2 expression and activity, reducing DNA damage and ROS production. Furthermore, we demonstrated that miR‐382‐5p inhibition impairs the proliferation of PMF CD34+ cells.

As a whole, miR‐382‐5p overexpression, leading to *SOD2* downregulation in HSPCs, could explain the overproduction of ROS and therefore the accumulation of oxidative DNA damage observed in PMF patients. As a consequence, DNA damage generates genomic instability that leads to the acquisition of new mutations allowing the clonal evolution and the disease progression (Marty *et al*., 2013).

One of the main features underlying PMF pathogenesis is the deregulation of the serum levels of pro‐inflammatory cytokines (Tefferi *et al*., 2011), which contribute to a state of chronic inflammation (Barosi, 2014; Skov *et al*., 2013). In particular, several findings have highlighted the key role of TGF‐β signaling in inflammation through the modulation of cytokine production, tumor migration, and angiogenesis (Pickup *et al*., 2013; Siegel and Massague, 2003). Moreover, TGF‐β1 has been reported to be a strong inducer of BM fibrosis (Agarwal *et al*., 2016), which is another important feature of PMF. Since it has been demonstrated that TGF‐β1 stimulates miR‐382‐5p expression during renal fibrosis (Kriegel *et al*., 2010), we investigated whether it could be able to regulate the miR‐382‐5p expression in normal CD34+ cells. Here we demonstrated that TGF‐β1 treatment enhances miR‐382‐5p expression and in turn suppresses SOD2 activity in normal CD34+ cells. According to our previous results, we highlighted an increase in ROS production after TGF‐β1 treatment, probably due to a reduction in SOD2 activity.

Finally, in order to further confirm the involvement of the axis TGF‐β1/miR‐382‐5p/*SOD2* in PMF pathogenesis, we inhibited TGF‐β1 pathway in PMF CD34+ cells using the TGF‐β‐receptor I kinase inhibitor galunisertib. Among the TGF‐β inhibitors, galunisertib preclinical efficacy has been already tested in different mouse models (Yue *et al*., 2017), demonstrating a strong effect in reversing or reducing BM fibrosis. Moreover, it has been demonstrated that galunisertib is a strong inhibitor of TGF‐β signaling in hematopoietic cells (Zhou *et al*., 2011). Here we showed that galunisertib is able to revert the effects induced by TGF‐β1 in both normal and PMF CD34+ cells. However, galunisertib alone reduces miR‐382‐5p expression and increases SOD2 activity only in PMF samples. Interestingly, galunisertib is also able to reduce ROS accumulation in PMF CD34+ cells. These results are in line with the evidence that TGF‐β1 signaling is a hyperactivated pathway in MPNs (Kota *et al*., 2008).

**Figure 9:**
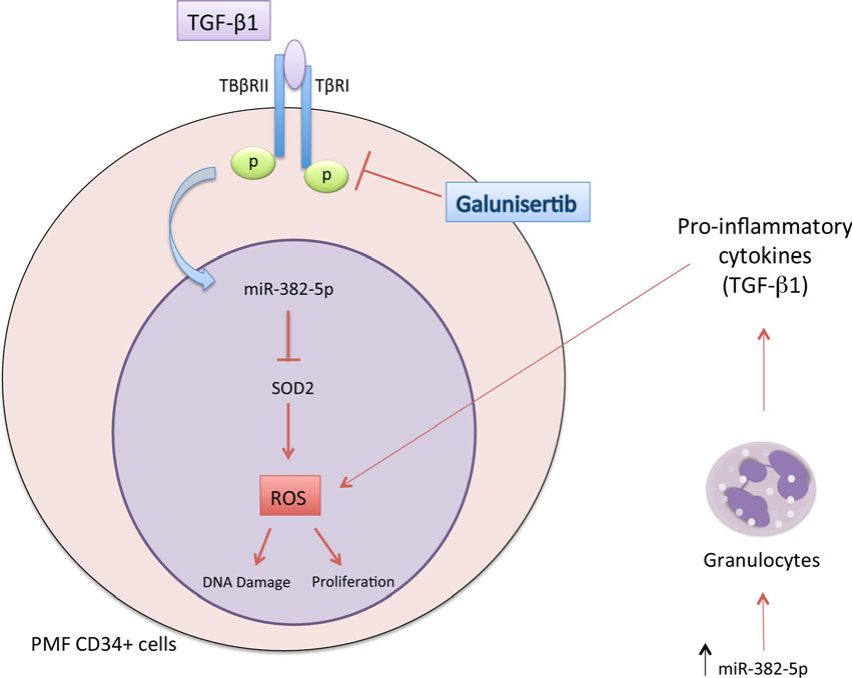
The axis TGF‐β1/miR‐382‐5p/SOD2. The axis TGF‐β1/miR‐382‐5p/SOD2 as mechanism underlying the overproduction of ROS in CD34+ PMF cells. ROS level could increase as a consequence of: (a) the release of pro‐inflammatory cytokines from granulocytes induced by miR‐382‐5p overexpression; (b) the enhanced expression of miR‐382‐5p, which reduces the expression of its target *SOD2*; (c) the upregulation of miR‐382‐5p by TGF‐β1. The TGF‐β‐receptor I kinase inhibitor galunisertib could inhibit the production of ROS by reducing the expression level of miR‐382‐5p and restoring SOD2 activity.

Our data support the idea that the chronic state of inflammation can itself promote ROS accumulation, which in turn generates more inflammation. Moreover, since we have already demonstrated that miR‐382‐5p stimulates the granulocyte lineage (Zini *et al*., 2016), we hypothesized that the increased release of pro‐inflammatory cytokines (i.e., TGF‐β1) from granulocytes themselves could activate the miR‐382‐5p/*SOD2* axis in PMF CD34+ cells sustaining the high oxidative stress state (Fig. 9).

In summary, we propose a new role for TGF‐β1 as a key mediator in ROS overproduction and we bring further evidence of its contribution in establishing a state of chronic inflammation. Strikingly, we identified a negative correlation between the expression level of *SOD2* and the grade of BM fibrosis in PMF, suggesting that ROS accumulation caused by SOD2 reduction could favor the TGF‐β1‐driven progression of fibrosis (Fig. S4).

Moreover, the reduction in ROS content induced by galunisertib in PMF CD34+ cells indicates that it should be an effective therapy to decrease the oxidative stress produced and sustained by the increased level of TGF‐β1 in PMF patients.

As a whole, our data suggest that the combination therapy of galunisertib with JAK2 inhibitors, which is currently the gold standard for PMF therapy, should represent a novel therapeutic option.

## 5 Conclusions

Here we describe a new role of TGF‐β1 as ROS inducer. Alongside its well‐known function in PMF fibrotic process, we demonstrated that TGF‐β1 increases the production of ROS through the modulation of the axis miR‐382‐5p/*SOD2*. Moreover, we shed light on the novel therapeutic potential of galunisertib, a TGF‐β1 inhibitor, in reducing ROS accumulation and oxidative stress in PMF CD34+ cells.

## Author contributions

CR, RZ, and RM designed the study and wrote the manuscript; RZ, ET, and SeRo performed microarray experiments; CR, SaRu, and EB performed miRNA overexpression and silencing experiments; CR and GB carried out luciferase reporter assays and qRT‐PCR; CR and ZP performed SOD2 activity assay, ROS detection, and DNA oxidation assays; CR, EG, and SS performed immunostaining analysis; AMV, PG, NB, GB, and VR enrolled patients.

## Supporting information


**Fig. S1.** miRNA transfection efficiency.

**Fig. S2.** SOD2 expression in CD34+ cells from PMF patients and healthy donors.

**Fig. S3.** Galunisertib (LY‐2157299) treatment in normal CD34+ cells.

**Fig. S4.** Fibrosis grade according to SOD2 expression level in PMF CD34+ cells.

**Table S1.** Deregulated genes upon miR‐382‐5p overexpression.
**Table S2**. List of miR‐382‐5p predicted targets.

## References

[mol212387-bib-0001] Agarwal A , Morrone K , Bartenstein M , Zhao ZJ , Verma A and Goel S (2016) Bone marrow fibrosis in primary myelofibrosis: pathogenic mechanisms and the role of TGF‐beta. Stem Cell Investig 3, 5. 10.3978/j.issn.2306-9759.2016.02.03 PMC4923632 27358897

[mol212387-bib-0002] Araki M , Yang Y , Masubuchi N , Hironaka Y , Takei H , Morishita S , Mizukami Y , Kan S , Shirane S , Edahiro Y *et al* (2016) Activation of the thrombopoietin receptor by mutant calreticulin in CALR‐mutant myeloproliferative neoplasms. Blood 127, 1307–1316. 26817954 10.1182/blood-2015-09-671172

[mol212387-bib-0003] Arber DA , Orazi A , Hasserjian R , Thiele J , Borowitz MJ , Le Beau MM , Bloomfield CD , Cazzola M and Vardiman JW (2016) The 2016 revision to the World Health Organization classification of myeloid neoplasms and acute leukemia. Blood 127, 2391–2405. 27069254 10.1182/blood-2016-03-643544

[mol212387-bib-0004] Barosi G (2014) An immune dysregulation in MPN. Curr Hematol Malig Rep 9, 331–339. 25139710 10.1007/s11899-014-0227-0

[mol212387-bib-0005] Bartel DP (2004) MicroRNAs: genomics, biogenesis, mechanism, and function. Cell 116, 281–297. 14744438 10.1016/s0092-8674(04)00045-5

[mol212387-bib-0006] Bianchi E , Zini R , Salati S , Tenedini E , Norfo R , Tagliafico E , Manfredini R and Ferrari S (2010) c‐myb supports erythropoiesis through the transactivation of KLF1 and LMO2 expression. Blood 116, e99–e110. 20686118 10.1182/blood-2009-08-238311

[mol212387-bib-0007] Bjorn ME and Hasselbalch HC (2015) The role of reactive oxygen species in myelofibrosis and related neoplasms. Mediators Inflamm 2015, 648090. 26538833 10.1155/2015/648090 PMC4619981

[mol212387-bib-0008] Bruchova H , Yoon D , Agarwal AM , Mendell J and Prchal JT (2007) Regulated expression of microRNAs in normal and polycythemia vera erythropoiesis. Exp Hematol 35, 1657–1667. 17976518 10.1016/j.exphem.2007.08.021 PMC2699372

[mol212387-bib-0009] Chachoua I , Pecquet C , El‐Khoury M , Nivarthi H , Albu RI , Marty C , Gryshkova V , Defour JP , Vertenoeil G , Ngo A *et al* (2016) Thrombopoietin receptor activation by myeloproliferative neoplasm associated calreticulin mutants. Blood 127, 1325–1335. 26668133 10.1182/blood-2015-11-681932

[mol212387-bib-0010] Chen E , Beer PA , Godfrey AL , Ortmann CA , Li J , Costa‐Pereira AP , Ingle CE , Dermitzakis ET , Campbell PJ and Green AR (2010) Distinct clinical phenotypes associated with JAK2V617F reflect differential STAT1 signaling. Cancer Cell 18, 524–535. 21074499 10.1016/j.ccr.2010.10.013 PMC2996868

[mol212387-bib-0011] Gloire G , Legrand‐Poels S and Piette J (2006) NF‐kappaB activation by reactive oxygen species: fifteen years later. Biochem Pharmacol 72, 1493–1505. 16723122 10.1016/j.bcp.2006.04.011

[mol212387-bib-0012] Guglielmelli P , Tozzi L , Pancrazzi A , Bogani C , Antonioli E , Ponziani V , Poli G , Zini R , Ferrari S , Manfredini R *et al* (2007a) MicroRNA expression profile in granulocytes from primary myelofibrosis patients. Exp Hematol 35, 1708–1718. 17976522 10.1016/j.exphem.2007.08.020

[mol212387-bib-0013] Guglielmelli P , Zini R , Bogani C , Salati S , Pancrazzi A , Bianchi E , Mannelli F , Ferrari S , Le Bousse‐Kerdiles MC , Bosi A *et al* (2007b) Molecular profiling of CD34 + cells in idiopathic myelofibrosis identifies a set of disease‐associated genes and reveals the clinical significance of Wilms’ tumor gene 1 (WT1). Stem Cells 25, 165–173. 16990584 10.1634/stemcells.2006-0351

[mol212387-bib-0014] Hasselbalch HC and Bjorn ME (2015) MPNs as inflammatory diseases: the evidence, consequences, and perspectives. Mediators Inflamm 2015, 102476. 26604428 10.1155/2015/102476 PMC4641200

[mol212387-bib-0015] Hitchler MJ , Oberley LW and Domann FE (2008) Epigenetic silencing of SOD2 by histone modifications in human breast cancer cells. Free Radic Biol Med 45, 1573–1580. 18845242 10.1016/j.freeradbiomed.2008.09.005 PMC2633123

[mol212387-bib-0016] Hole PS , Pearn L , Tonks AJ , James PE , Burnett AK , Darley RL and Tonks A (2010) Ras‐induced reactive oxygen species promote growth factor‐independent proliferation in human CD34 + hematopoietic progenitor cells. Blood 115, 1238–1246. 20007804 10.1182/blood-2009-06-222869

[mol212387-bib-0017] Humpe A , Beck C , Schoch R , Kneba M and Horst HA (2005) Establishment and optimization of a flow cytometric method for evaluation of viability of CD34 + cells after cryopreservation and comparison with trypan blue exclusion staining. Transfusion 45, 1208–1213. 15987368 10.1111/j.1537-2995.2005.00174.x

[mol212387-bib-0018] Hurt EM , Thomas SB , Peng B and Farrar WL (2007) Integrated molecular profiling of SOD2 expression in multiple myeloma. Blood 109, 3953–3962. 17192397 10.1182/blood-2006-07-035162 PMC1874573

[mol212387-bib-0019] Inoue M , Sato EF , Nishikawa M , Park AM , Kira Y , Imada I and Utsumi K (2003) Mitochondrial generation of reactive oxygen species and its role in aerobic life. Curr Med Chem 10, 2495–2505. 14529465 10.2174/0929867033456477

[mol212387-bib-0020] Irizarry RA , Hobbs B , Collin F , Beazer‐Barclay YD , Antonellis KJ , Scherf U and Speed TP (2003) Exploration, normalization, and summaries of high density oligonucleotide array probe level data. Biostatistics 4, 249–264. 12925520 10.1093/biostatistics/4.2.249

[mol212387-bib-0021] Jongen‐Lavrencic M , Sun SM , Dijkstra MK , Valk PJ and Lowenberg B (2008) MicroRNA expression profiling in relation to the genetic heterogeneity of acute myeloid leukemia. Blood 111, 5078–5085. 18337557 10.1182/blood-2008-01-133355

[mol212387-bib-0022] Klampfl T , Gisslinger H , Harutyunyan AS , Nivarthi H , Rumi E , Milosevic JD , Them NC , Berg T , Gisslinger B , Pietra D *et al* (2013) Somatic mutations of calreticulin in myeloproliferative neoplasms. N Engl J Med 369, 2379–2390. 24325356 10.1056/NEJMoa1311347

[mol212387-bib-0023] Kota J , Caceres N and Constantinescu SN (2008) Aberrant signal transduction pathways in myeloproliferative neoplasms. Leukemia 22, 1828–1840. 18769448 10.1038/leu.2008.236

[mol212387-bib-0024] Kriegel AJ , Fang Y , Liu Y , Tian Z , Mladinov D , Matus IR , Ding X , Greene AS and Liang M (2010) MicroRNA‐target pairs in human renal epithelial cells treated with transforming growth factor beta 1: a novel role of miR‐382. Nucleic Acids Res 38, 8338–8347. 20716515 10.1093/nar/gkq718 PMC3001085

[mol212387-bib-0025] Leask A and Abraham DJ (2004) TGF‐beta signaling and the fibrotic response. FASEB J 18, 816–827. 15117886 10.1096/fj.03-1273rev

[mol212387-bib-0026] Li Z , Lu J , Sun M , Mi S , Zhang H , Luo RT , Chen P , Wang Y , Yan M , Qian Z *et al* (2008) Distinct microRNA expression profiles in acute myeloid leukemia with common translocations. Proc Natl Acad Sci USA 105, 15535–15540. 18832181 10.1073/pnas.0808266105 PMC2563085

[mol212387-bib-0027] Marty C , Lacout C , Droin N , Le Couedic JP , Ribrag V , Solary E , Vainchenker W , Villeval JL and Plo I (2013) A role for reactive oxygen species in JAK2 V617F myeloproliferative neoplasm progression. Leukemia 27, 2187–2195. 23558526 10.1038/leu.2013.102

[mol212387-bib-0028] Marty C , Pecquet C , Nivarthi H , El‐Khoury M , Chachoua I , Tulliez M , Villeval JL , Raslova H , Kralovics R , Constantinescu SN *et al* (2016) Calreticulin mutants in mice induce an MPL‐dependent thrombocytosis with frequent progression to myelofibrosis. Blood 127, 1317–1324. 26608331 10.1182/blood-2015-11-679571

[mol212387-bib-0029] Nangalia J , Massie CE , Baxter EJ , Nice FL , Gundem G , Wedge DC , Avezov E , Li J , Kollmann K , Kent DG *et al* (2013) Somatic CALR mutations in myeloproliferative neoplasms with nonmutated JAK2. N Engl J Med 369, 2391–2405. 24325359 10.1056/NEJMoa1312542 PMC3966280

[mol212387-bib-0030] Norfo R , Zini R , Pennucci V , Bianchi E , Salati S , Guglielmelli P , Bogani C , Fanelli T , Mannarelli C , Rosti V *et al* (2014) miRNA‐mRNA integrative analysis in primary myelofibrosis CD34 + cells: role of miR‐155/JARID2 axis in abnormal megakaryopoiesis. Blood 124, e21–e32. 25097177 10.1182/blood-2013-12-544197 PMC4186546

[mol212387-bib-0031] Oberley LW (2005) Mechanism of the tumor suppressive effect of MnSOD overexpression. Biomed Pharmacother 59, 143–148. 15862707 10.1016/j.biopha.2005.03.006

[mol212387-bib-0032] Pardanani AD , Levine RL , Lasho T , Pikman Y , Mesa RA , Wadleigh M , Steensma DP , Elliott MA , Wolanskyj AP , Hogan WJ *et al* (2006) MPL515 mutations in myeloproliferative and other myeloid disorders: a study of 1182 patients. Blood 108, 3472–3476. 16868251 10.1182/blood-2006-04-018879

[mol212387-bib-0033] Pickup M , Novitskiy S and Moses HL (2013) The roles of TGFbeta in the tumour microenvironment. Nat Rev Cancer 13, 788–799. 24132110 10.1038/nrc3603 PMC4025940

[mol212387-bib-0034] Siegel PM and Massague J (2003) Cytostatic and apoptotic actions of TGF‐beta in homeostasis and cancer. Nat Rev Cancer 3, 807–821. 14557817 10.1038/nrc1208

[mol212387-bib-0035] Skov V , Riley CH , Thomassen M , Larsen TS , Jensen MK , Bjerrum OW , Kruse TA and Hasselbalch HC (2013) Whole blood transcriptional profiling reveals significant down‐regulation of human leukocyte antigen class I and II genes in essential thrombocythemia, polycythemia vera and myelofibrosis. Leuk Lymphoma 54, 2269–2273. 23302045 10.3109/10428194.2013.764417

[mol212387-bib-0036] Sobecki M , Mrouj K , Camasses A , Parisis N , Nicolas E , Lleres D , Gerbe F , Prieto S , Krasinska L , David A *et al* (2016) The cell proliferation antigen Ki‐67 organises heterochromatin. Elife 5, e13722. 26949251 10.7554/eLife.13722 PMC4841783

[mol212387-bib-0037] Szeto HH (2006) Mitochondria‐targeted peptide antioxidants: novel neuroprotective agents. AAPS J 8, E521–E531. 17025271 10.1208/aapsj080362 PMC2761060

[mol212387-bib-0038] Tefferi A (2005) Pathogenesis of myelofibrosis with myeloid metaplasia. J Clin Oncol 23, 8520–8530. 16293880 10.1200/JCO.2004.00.9316

[mol212387-bib-0039] Tefferi A , Vaidya R , Caramazza D , Finke C , Lasho T and Pardanani A (2011) Circulating interleukin (IL)‐8, IL‐2R, IL‐12, and IL‐15 levels are independently prognostic in primary myelofibrosis: a comprehensive cytokine profiling study. J Clin Oncol 29, 1356–1363. 21300928 10.1200/JCO.2010.32.9490

[mol212387-bib-0040] Vaidya R , Gangat N , Jimma T , Finke CM , Lasho TL , Pardanani A and Tefferi A (2012) Plasma cytokines in polycythemia vera: phenotypic correlates, prognostic relevance, and comparison with myelofibrosis. Am J Hematol 87, 1003–1005. 22965887 10.1002/ajh.23295

[mol212387-bib-0041] Vainchenker W and Kralovics R (2017) Genetic basis and molecular pathophysiology of classical myeloproliferative neoplasms. Blood 129, 667–679. 28028029 10.1182/blood-2016-10-695940

[mol212387-bib-0042] Van Remmen H , Ikeno Y , Hamilton M , Pahlavani M , Wolf N , Thorpe SR , Alderson NL , Baynes JW , Epstein CJ , Huang TT *et al* (2003) Life‐long reduction in MnSOD activity results in increased DNA damage and higher incidence of cancer but does not accelerate aging. Physiol Genomics 16, 29–37. 14679299 10.1152/physiolgenomics.00122.2003

[mol212387-bib-0043] Vardiman JW , Thiele J , Arber DA , Brunning RD , Borowitz MJ , Porwit A , Harris NL , Le Beau MM , Hellstrom‐Lindberg E , Tefferi A *et al* (2009) The 2008 revision of the World Health Organization (WHO) classification of myeloid neoplasms and acute leukemia: rationale and important changes. Blood 114, 937–951. 19357394 10.1182/blood-2009-03-209262

[mol212387-bib-0044] Vener C , Novembrino C , Catena FB , Fracchiolla NS , Gianelli U , Savi F , Radaelli F , Fermo E , Cortelezzi A , Lonati S *et al* (2010) Oxidative stress is increased in primary and post‐polycythemia vera myelofibrosis. Exp Hematol 38, 1058–1065. 20655352 10.1016/j.exphem.2010.07.005

[mol212387-bib-0045] Wang R , Yin C , Li XX , Yang XZ , Yang Y , Zhang MY , Wang HY and Zheng XF (2016) Reduced SOD2 expression is associated with mortality of hepatocellular carcinoma patients in a mutant p53‐dependent manner. Aging (Albany NY) 8, 1184–1200. 27221200 10.18632/aging.100967 PMC4931826

[mol212387-bib-0046] Waris G and Ahsan H (2006) Reactive oxygen species: role in the development of cancer and various chronic conditions. J Carcinog 5, 14. 16689993 10.1186/1477-3163-5-14 PMC1479806

[mol212387-bib-0047] Whitfield ML , George LK , Grant GD and Perou CM (2006) Common markers of proliferation. Nat Rev Cancer 6, 99–106. 16491069 10.1038/nrc1802

[mol212387-bib-0048] Yahata T , Takanashi T , Muguruma Y , Ibrahim AA , Matsuzawa H , Uno T , Sheng Y , Onizuka M , Ito M , Kato S *et al* (2011) Accumulation of oxidative DNA damage restricts the self‐renewal capacity of human hematopoietic stem cells. Blood 118, 2941–2950. 21734240 10.1182/blood-2011-01-330050

[mol212387-bib-0049] Yue L , Bartenstein M , Zhao W , Ho WT , Han Y , Murdun C , Mailloux AW , Zhang L , Wang X , Budhathoki A *et al* (2017) Efficacy of ALK5 inhibition in myelofibrosis. JCI Insight 2, e90932. 28405618 10.1172/jci.insight.90932 PMC5374075

[mol212387-bib-0050] Zhan H , Cardozo C and Raza A (2013) MicroRNAs in myeloproliferative neoplasms. Br J Haematol 161, 471–483. 23432162 10.1111/bjh.12276 PMC5181108

[mol212387-bib-0051] Zheng S , Zhong ZM , Qin S , Chen GX , Wu Q , Zeng JH , Ye WB , Li W , Yuan K , Yao L *et al* (2013) Advanced oxidation protein products induce inflammatory response in fibroblast‐like synoviocytes through NADPH oxidase‐dependent activation of NF‐kappaB. Cell Physiol Biochem 32, 972–985. 24107363 10.1159/000354500

[mol212387-bib-0052] Zhou L , McMahon C , Bhagat T , Alencar C , Yu Y , Fazzari M , Sohal D , Heuck C , Gundabolu K , Ng C *et al* (2011) Reduced SMAD7 leads to overactivation of TGF‐beta signaling in MDS that can be reversed by a specific inhibitor of TGF‐beta receptor I kinase. Cancer Res 71, 955–963. 21189329 10.1158/0008-5472.CAN-10-2933 PMC3032816

[mol212387-bib-0053] Zini R , Guglielmelli P , Pietra D , Rumi E , Rossi C , Rontauroli S , Genovese E , Fanelli T , Calabresi L , Bianchi E *et al* (2017) CALR mutational status identifies different disease subtypes of essential thrombocythemia showing distinct expression profiles. Blood Cancer J 7, 638. 29217833 10.1038/s41408-017-0010-2 PMC5802509

[mol212387-bib-0054] Zini R , Rossi C , Norfo R , Pennucci V , Barbieri G , Ruberti S , Rontauroli S , Salati S , Bianchi E and Manfredini R (2016) miR‐382‐5p controls hematopoietic stem cell differentiation through the downregulation of MXD1. Stem Cells Dev 25, 1433–1443. 27520398 10.1089/scd.2016.0150

