# Supplementary material for: Role of TGF‐β1/miR‐382‐5p/SOD2 axis in the induction of oxidative stress in CD34+ cells from primary myelofibrosis
Source: Mol Oncol. 2018 Nov 16;12(12):2102–23. doi: 10.1002/1878-0261.12387 (PMC6275274; doi:10.1002/1878-0261.12387)
Supplement: Supplementary file 1 — Fig. S1. miRNA transfection efficiency. [file MOL2-12-2102-s001.pdf]

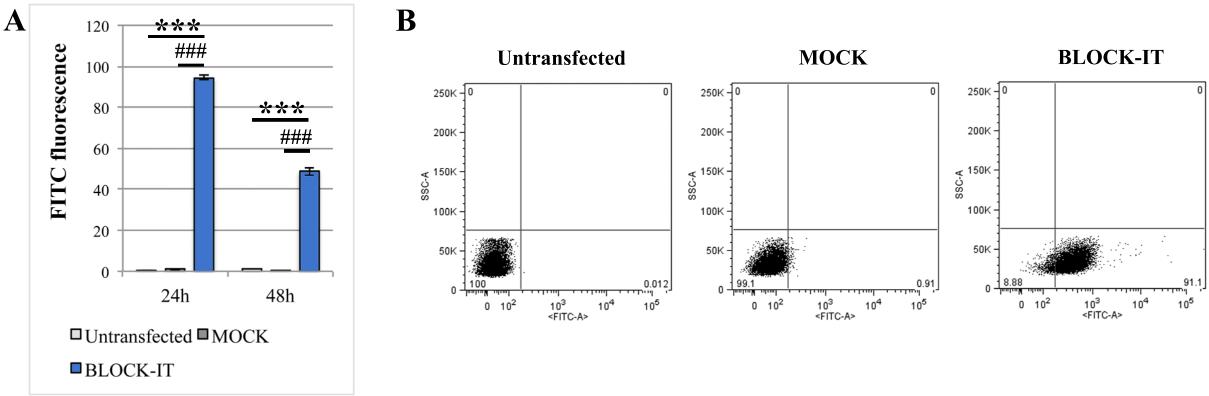

Figure S1

Figure S1: miRNA transfection efficiency. (A) BLOCK-IT Fluorescent Oligo was used to estimate the miRNA transfection efficiency in CD34+ cells. CD34+ cells underwent two nucleofection every 24 hours with 0.214 nmoles BLOCK-IT fluorescent oligo using the same protocol as for miRNA transfection. FITC fluorescence was detected by flow cytometry at 24 and 48 hours after the last nucleofection. **(B)** Representative flow cytometry dot plot showing FITC fluorescence in BLOCK-IT transfected cells compared to un-transfected and MOCK-transfected samples at 24 hours after the last nucleo-fection. 2-tailed Student t test: ***, $P < 0.001$ versus Untransfected Sample; ###, $P < 0.001$ versus MOCK-transfected cells.

Abbreviations: h, hours; FITC, Fluorescein isothiocyanate; SSC, Side scatter
