# Supplementary material for: Role of TGF‐β1/miR‐382‐5p/SOD2 axis in the induction of oxidative stress in CD34+ cells from primary myelofibrosis
Source: Mol Oncol. 2018 Nov 16;12(12):2102–23. doi: 10.1002/1878-0261.12387 (PMC6275274; doi:10.1002/1878-0261.12387)
Supplement: Supplementary file 2 — Fig. S2. SOD2 expression in CD34+ cells from PMF patients and healthy donors. [file MOL2-12-2102-s002.pdf]

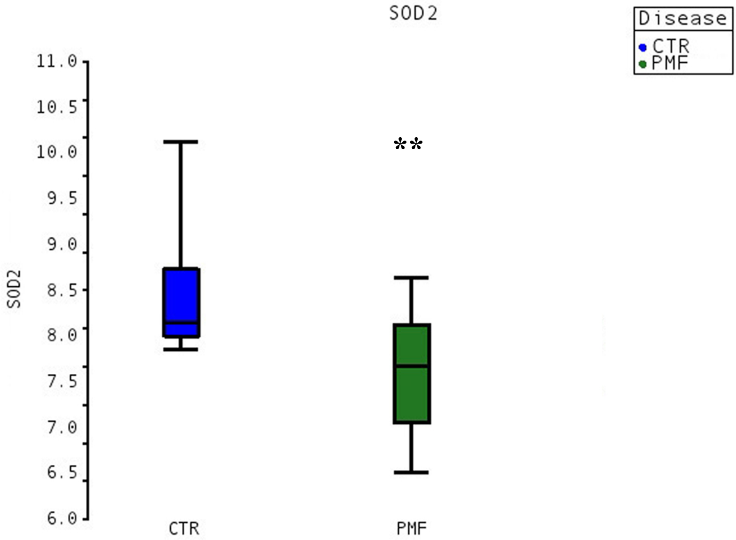

Figure S2

Figure S2: SOD2 expression in CD34+ cells from PMF patients and healthy donors.
SOD2 expression level was assessed by micro-array analysis in 42 PMF patients and 16 healthy donors by means of Affymetrix platform as previously described (Norfo et al., 2014).
Mann–Whitney U test: **, P < .01 versus CTR, 2-tailed student t-test.
Abbreviations: SOD2, superoxide dismutase 2; CTR, healthy donors; PMF, primary myelofibrosis.
