# Supplementary material for: Role of TGF‐β1/miR‐382‐5p/SOD2 axis in the induction of oxidative stress in CD34+ cells from primary myelofibrosis
Source: Mol Oncol. 2018 Nov 16;12(12):2102–23. doi: 10.1002/1878-0261.12387 (PMC6275274; doi:10.1002/1878-0261.12387)
Supplement: Supplementary file 3 — Fig. S3. Galunisertib (LY‐2157299) treatment in normal CD34+ cells. [file MOL2-12-2102-s003.pdf]

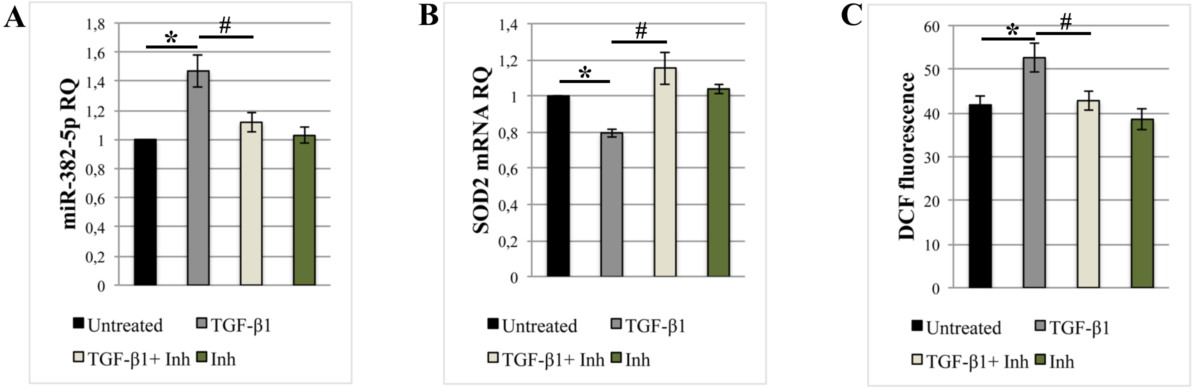

Figure S3

Figure S3: Galunisertib (LY-2157299) treatment in normal CD34+ cells. CB CD34+ cells were treated with TGF-β1 or galunisertib (Inh) alone or TGF-β1 and galunisertib (TGF-β1+Inh) in combination. **(A-B)** qRT-PCR analysis was performed to evaluate the expression level of miR-382-5p and SOD2 at 24 hours after treatments. U6 snRNA and GAPDH were used as internal controls. Untreated cells were used to normalize the data. **(C)** Level of ROS production measured by DCF staining 24 hours after TGF-β1/TGF-β1+ Inh/Inh treatments. The results are representative of three independent experiments and are expressed as the mean ± SEM. 2-tailed Student t test: **, P<.01 versus untreated cells; *, P<.05 versus untreated cells. #, P<.05 versus TGF-β1 treated cells.

Abbreviations: RQ, relative quantity; SOD2, superoxide dismutase 2; TGF-β1, transforming growth factor β1; Inh, inhibitor (galunisertib); DCF, dichrolo-fluorescein.
