# Supplementary material for: Role of TGF‐β1/miR‐382‐5p/SOD2 axis in the induction of oxidative stress in CD34+ cells from primary myelofibrosis
Source: Mol Oncol. 2018 Nov 16;12(12):2102–23. doi: 10.1002/1878-0261.12387 (PMC6275274; doi:10.1002/1878-0261.12387)
Supplement: Supplementary file 4 — Fig. S4. Fibrosis grade according to SOD2 expression level in PMF CD34+ cells. [file MOL2-12-2102-s004.pdf]

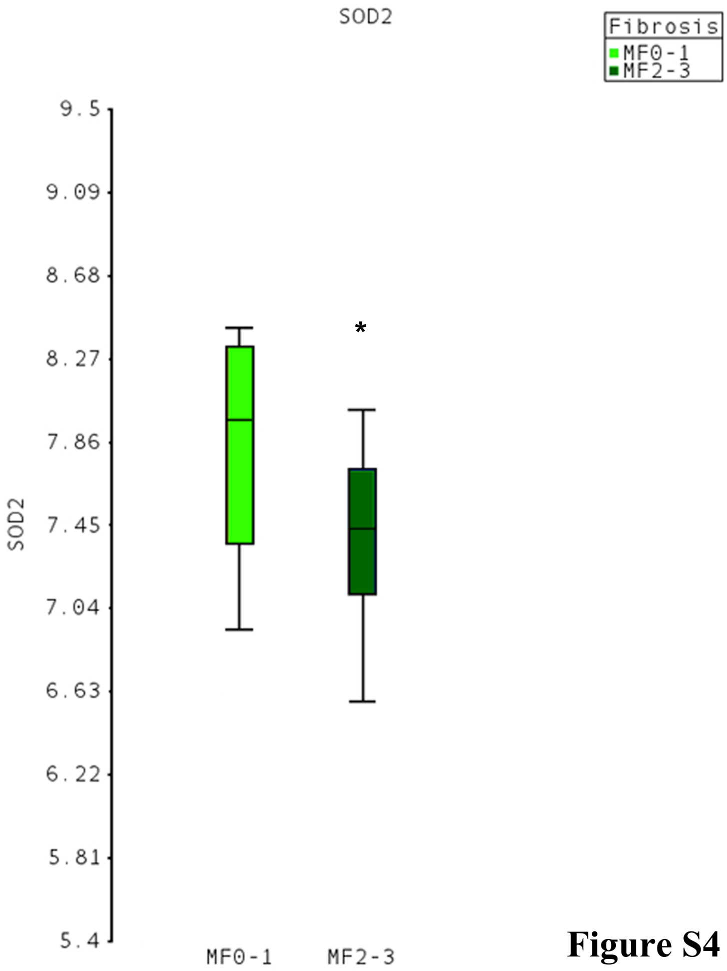

Figure S4

Figure S4: Fibrosis grade according to SOD2 expression level in PMF CD34+ cells. SOD2 expression level in CD34+ cells from PMF patients at different stage of bone marrow (BM) fibrosis. PMF patients were split in two different stages according to BM fibrosis grade: prefibrotic (MF-0/1, n=11) and overt fibrotic (MF-2/3, n=66). Gene expression level was measured by microarray analysis by means of Affymetrix platform. Mann-Whitney U test: *, $P < .05$ versus MF 0-1, 2-tailed student t-test.
