# Supplementary material for: Role of TGF‐β1/miR‐382‐5p/SOD2 axis in the induction of oxidative stress in CD34+ cells from primary myelofibrosis
Source: Mol Oncol. 2018 Nov 16;12(12):2102–23. doi: 10.1002/1878-0261.12387 (PMC6275274; doi:10.1002/1878-0261.12387)
Supplement: Supplementary file 5 — Table S1. Deregulated genes upon miR‐382‐5p overexpression. Table S2. List of miR‐382‐5p predicted targets. [file MOL2-12-2102-s005.pdf]

Table S1: Deregulated genes upon miR-382-5p overexpression.

List of 75 down-regulated genes in miR-382-5p-overexpressing cells compared to negative control.

Gene Symbol	Gene Title	Signal Log Ratio (miR-382-5p vs mimic-Neg)	T test
ALOX5AP	arachidonate 5-lipoxygenase-activating protein	-0,40409	0,007048777
ANKS1A	ankyrin repeat and sterile alpha motif domain containing 1A	-0,442766667	0,007807842
APOL3	apolipoprotein L, 3	-0,47642	0,01279588
ARHGEF12	Rho guanine nucleotide exchange factor (GEF) 12	-0,465093333	0,011533218
ARPC2	actin related protein 2/3 complex, subunit 2, 34kDa	-0,4697	0,020684003
BEX2	brain expressed X-linked 2	-0,555523333	0,030498322
BTBD7	BTB (POZ) domain containing 7	-0,461716667	0,048814394
C11orf58	chromosome 11 open reading frame 58	-0,59299	0,029231099
C1orf162	chromosome 1 open reading frame 162	-0,602153333	0,01243519
C2orf42	chromosome 2 open reading frame 42	-0,478276667	0,015402691
CBS	cystathionine-beta-synthase	-0,447813333	0,045051282
CCL1	chemokine (C-C motif) ligand 1	-0,485036667	0,004869042
CCL4 /// CCL4L1 /// CCL4L2	chemokine (C-C motif) ligand 4	-0,627856667	0,027576819
CD302 /// LY75-CD302	CD302 molecule /// LY75-CD302 readthrough	-0,507723333	0,017847814
CHN2	chimerin 2	-0,490196667	0,009238229
CNOT6	CCR4-NOT transcription complex, subunit 6	-0,51234	0,031199364
CRHBP	corticotropin releasing hormone binding protein	-0,473923333	0,003281025
DDIT4	DNA-damage-inducible transcript 4	-0,423593333	0,002345132
DICER1	dicer 1, ribonuclease type III	-0,763223333	0,018771809
EFS	embryonal Fyn-associated substrate	-0,416136667	0,012609438
FBXO28	F-box protein 28	-0,775446667	0,008227622
FPR2	formyl peptide receptor 2	-0,569036667	0,00932636
FUNDC1	FUN14 domain containing 1	-0,47942	0,012941108

FZD6	frizzled family receptor 6	-0,431593333	0,028777487
GMFB	glia maturation factor, beta	-0,403356667	0,014894292
GOLM1	golgi membrane protein 1	-0,977196667	0,012857854
GUCY1A3	guanylate cyclase 1, soluble, alpha 3	-0,560776667	0,035150148
IFI6	interferon, alpha-inducible protein 6	-0,400153333	0,017314535
IFITM1	interferon induced transmembrane protein 1	-0,609186667	0,021515818
IGFBP5	insulin-like growth factor binding protein 5	-0,407253333	0,016707207
IGFLR1	IGF-like family receptor 1	-0,41164	0,001412494
KAT7	K(lysine) acetyltransferase 7	-0,485053333	0,004341322
LGALS12	lectin, galactoside-binding, soluble, 12	-0,578686667	0,04615049
LIF	leukemia inhibitory factor	-0,632066667	0,039122947
LILRA2	leukocyte immunoglobulin-like receptor, subfamily A (with TM domain), member 2	-0,417973333	0,022312236
LRP12	low density lipoprotein receptor-related protein 12	-0,700556667	0,011459215
LY96	lymphocyte antigen 96	-0,435026667	0,022150319
MMP12	matrix metallopeptidase 12 (macrophage elastase)	-1,02581	0,033374219
MX1	myxovirus (influenza virus) resistance 1, interferon-inducible protein p78 (mouse)	-0,4104	0,04811591
NDN	necdin, melanoma antigen (MAGE) family member	-1,54012	0,026710803
NFYC	nuclear transcription factor Y, gamma	-0,404473333	0,030534544
NLK	nemo-like kinase	-0,422136667	0,020463942
OLR1	oxidized low density lipoprotein (lectin-like) receptor 1	-0,43467	0,039323473
PACSIN2	protein kinase C and casein kinase substrate in neurons 2	-0,713846667	0,045984626
PAIP1	poly(A) binding protein interacting protein 1	-0,750683333	0,047111279
PDE4B	phosphodiesterase 4B, cAMP-specific	-0,47083	0,040989255
PPP3CC	protein phosphatase 3, catalytic subunit, gamma isozyme	-0,490716667	0,018165869
PSPH	phosphoserine phosphatase	-0,51363	0,003928957
QPCT	glutaminyl-peptide cyclotransferase	-0,548973333	0,021988113
RAP1B	RAP1B, member of RAS oncogene family	-0,711433333	0,038132162
RARRES3	retinoic acid receptor responder (tazarotene induced) 3	-0,670046667	0,025913496
RNASET2	ribonuclease T2	-0,417123333	0,008734671

RPIA	ribose 5-phosphate isomerase A	-0,955816667	0,041496062
S100A8	S100 calcium binding protein A8	-0,865343333	0,033142428
S100A9	S100 calcium binding protein A9	-0,737136667	0,02951256
SEL1L3	sel-1 suppressor of lin-12-like 3 (C. elegans)	-0,461963333	0,004257096
SEMA4D	sema domain, immunoglobulin domain (Ig), transmembrane domain (TM) and short cytoplasmic domain, (semaphorin) 4D	-0,407096667	0,011311337
SETD8	SET domain containing (lysine methyltransferase) 8	-0,448463333	0,001683985
SFR1	SWI5-dependent recombination repair 1	-0,592946667	0,022315143
SGTB	small glutamine-rich tetratricopeptide repeat (TPR)-containing, beta	-0,46603	0,032474958
SLAIN1	SLAIN motif family, member 1	-1,38505	0,017686941
SLC25A30	solute carrier family 25, member 30	-0,679313333	0,048903852
SOD2	superoxide dismutase 2, mitochondrial	-0,46748	0,021077249
SOWAHC	sosondowah ankyrin repeat domain family member C	-0,533563333	0,047674992
SPATS2	spermatogenesis associated, serine-rich 2	-0,565846667	0,025631927
SPEN	spen homolog, transcriptional regulator (Drosophila)	-0,423666667	0,017769008
STXBP6	syntaxin binding protein 6 (amisyn)	-0,565453333	0,023740135
TBL1X	transducin (beta)-like 1X-linked	-0,427786667	0,010834015
TNPO1	transportin 1	-0,575273333	0,008218385
TPM1	tropomyosin 1 (alpha)	-0,48008	0,006711364
TREM2	triggering receptor expressed on myeloid cells 2	-0,59986	0,044112095
TWSG1	twisted gastrulation homolog 1 (Drosophila)	-0,918916667	0,043313516
UBE3B	ubiquitin protein ligase E3B	-0,616476667	0,022822751
XAF1	XIAP associated factor 1	-0,5063	0,01859167
ZNF318	zinc finger protein 318	-0,54503	0,018727607

List of 22 up-regulated genes in miR-382-5p-overexpressing cells compared to negative control.

Gene symbol	Gene Title	Signal Log Ratio (miR-382-5p vs mimic-Neg)	T test
AKR1C1	aldo-keto reductase family 1, member C1	0,590946667	0,007832206
AKR1C1 /// AKR1C2	aldo-keto reductase family 1, member C1 /// aldo-keto reductase family 1, member C2	0,43057	0,022809987
B3GAT2	beta-1,3-glucuronyltransferase 2 (glucuronosyltransferase S)	0,42743	0,018827358
DNMT3B	DNA (cytosine-5-)-methyltransferase 3 beta	0,42683	0,00613001
FGFBP3	fibroblast growth factor binding protein 3	0,44413	0,020009541
ISOC1	isochorismatase domain containing 1	0,40284	0,033790278
LACE1	lactation elevated 1	0,488276667	0,005984658
LZTFL1	leucine zipper transcription factor-like 1	1,03421	0,018696299
MNS1	meiosis-specific nuclear structural 1	0,553806667	0,008655918
MZT1	mitotic spindle organizing protein 1	0,40427	0,035884415
NDUFS3 /// PTPMT1	NADH dehydrogenase (ubiquinone) Fe-S protein 3, 30kDa (NADH-coenzyme Q reductase) /// protein tyrosine phosphatase, mitochondrial 1	0,668153333	0,016440076
NPTX1	neuronal pentraxin I	0,63536	0,033274224
PTPMT1	protein tyrosine phosphatase, mitochondrial 1	0,492603333	0,007901588
RFK	riboflavin kinase	0,434126667	0,002377822
RHAG	Rh-associated glycoprotein	0,477416667	0,022993006
SIPA1L2	signal-induced proliferation-associated 1 like 2	0,44156	0,004016757
SNCA	synuclein, alpha (non A4 component of amyloid precursor)	0,454853333	0,017148409
SPG20	spastic paraplegia 20 (Troyer syndrome)	0,47802	0,029118786
SPTA1	spectrin, alpha, erythrocytic 1 (elliptocytosis 2)	0,53646	0,020075122
TAPT1	transmembrane anterior posterior transformation 1	0,68706	0,00352443
TMEM65	transmembrane protein 65	0,45154	0,037846359
TOMM20	translocase of outer mitochondrial membrane 20 homolog (yeast)	0,447566667	0,025778674

Table S2: List of miR-382-5p predicted targets. List of down-regulated genes upon mir-382-5p overexpression and predicted target according to TargetScanHuman Database (release 7.0).

Gene Symbol	Gene Title	Signal Log Ratio (miR-382-5p vs. mimic-Neg)	Target Scan Human (context++ score)	Site
ANKS1A	ankyrin repeat and sterile alpha motif domain containing 1A	-0,442766667	-0,03	conserved
ARHGEF12	Rho guanine nucleotide exchange factor (GEF) 12	-0,465093333	-0,02	conserved
ARPC2	actin related protein 2/3 complex, subunit 2, 34kDa	-0,4697	-0,19	conserved
BTBD7	BTB (POZ) domain containing 7	-0,461716667	-0,1	conserved
C11orf58	chromosome 11 open reading frame 58	-0,59299	-0,17	poorly conserved
C2orf42	chromosome 2 open reading frame 42	-0,478276667	-0,2	poorly conserved
CD302 /// LY75-CD302	CD302 molecule /// LY75-CD302 readthrough	-0,507723333	-0,1	poorly conserved
CNOT6	CCR4-NOT transcription complex, subunit 6	-0,51234	-0,09	poorly conserved
CRHBP	corticotropin releasing hormone binding protein	-0,473923333	-0,18	poorly conserved
DICER1	dicer 1, ribonuclease type III	-0,763223333	-0,03	conserved
FBXO28	F-box protein 28	-0,775446667	-0,36	conserved
FUNDC1	FUN14 domain containing 1	-0,47942	-0,42	poorly conserved
FZD6	frizzled family receptor 6	-0,431593333	-0,11	poorly conserved
GMFB	glia maturation factor, beta	-0,403356667	-0,13	poorly conserved
GOLM1	golgi membrane protein 1	-0,977196667	-0,23	poorly conserved
LRP12	low density lipoprotein receptor-related protein 12	-0,700556667	-0,12	conserved/poorly conserved
NLK	nemo-like kinase	-0,422136667	-0,03	conserved
PACSIN2	protein kinase C and casein kinase substrate in neurons 2	-0,713846667	-0,09	poorly conserved
PPP3CC	protein phosphatase 3, catalytic subunit, gamma isozyme	-0,490716667	-0,2	poorly conserved (2)
PSPH	phosphoserine phosphatase	-0,51363	-0,35	conserved/poorly conserved
RPIA	ribose 5-phosphate isomerase A	-0,955816667	-0,02	poorly conserved
SETD8	SET domain containing (lysine methyltransferase) 8	-0,448463333	-0,17	conserved

SFR1	SWI5-dependent recombination repair 1	-0,592946667	-0,47	conserved
SGTB	small glutamine-rich tetratricopeptide repeat (TPR)-containing, beta	-0,46603	-0,14	conserved
SLAIN1	SLAIN motif family, member 1	-1,38505	-0,32	conserved
SLC25A30	solute carrier family 25, member 30	-0,679313333	-0,19	poorly conserved
SOD2	superoxide dismutase 2, mitochondrial	-0,46748	-0,59	poorly conserved (3)
SOWAHC	sosondowah ankyrin repeat domain family member C	-0,533563333	-0,01	poorly conserved
TNPO1	transportin 1	-0,575273333	-0,08	poorly conserved (2)
TREM2	triggering receptor expressed on myeloid cells 2	-0,59986	-0,17	poorly conserved
TWSG1	twisted gastrulation homolog 1 (Drosophila)	-0,918916667	-0,22	poorly conserved (2)
XAF1	XIAP associated factor 1	-0,5063	-0,04	poorly conserved
ZNF318	zinc finger protein 318	-0,54503	-0,27	poorly conserved
